# Users’ experience of frameworks to support evidence-informed decision-making in public health: a scoping review

**DOI:** 10.2807/1560-7917.ES.2025.30.19.2400184

**Published:** 2025-05-15

**Authors:** Javier Bracchiglione, Yang Song, Jose F Meneses-Echávez, Helena de Carvalho Gomes, Barbara Albiger, Ivan Solà, David Rigau, Pablo Alonso-Coello

**Affiliations:** 1 Institut de Recerca Sant Pau (IR SANT PAU), Barcelona, Spain; 2 Centro Cochrane Iberoamericano, Barcelona, Spain; 3 Centro de Investigación Biomédica en Red de Epidemiología y Salud Pública, Instituto de Salud Carlos III, Madrid, Spain; 4 Interdisciplinary Centre for Health Studies (CIESAL), Universidad de Valparaíso, Viña del Mar, Chile; 5 School of Medicine, Chinese University of Hong Kong, Shenzhen, China; 6 Facultad de Cultura Física, Deporte, y Recreación, Universidad Santo Tomás, Bogotá, Colombia; 7 Norwegian Institute of Public Health, Oslo, Norway; 8 European Centre for Disease Prevention and Control (ECDC), Stockholm, Sweden

**Keywords:** infections, decision-making, health planning guidelines, public health, prevention and control

## Abstract

**Background:**

Evidence-informed decision-making in public health (PH) is a complex process requiring the consideration of multiple perspectives and contextual factors. Evidence-to-decision (EtD) frameworks are structured approaches aiming to improve decision-making by considering critical criteria, but users’ experience has not been systematically synthesised.

**Aim:**

We aim to summarise users’ experiences of EtD frameworks used for PH.

**Methods:**

As part of a broader scoping review, we identified 15 EtD frameworks for PH decision-making. We searched MEDLINE and Health Systems Evidence, conducted a hand search and citation search strategy for documents reporting users’ experience of EtD frameworks and surveyed key stakeholders. We conducted a descriptive thematic synthesis, identifying main barriers and facilitators, complementing with surveys to relevant stakeholders.

**Results:**

We identified 12 studies reporting users’ experience of two EtD frameworks: Grading of Recommendations Assessment, Development and Evaluation (n = 9) and World Health Organization INTEGRATe Evidence (n = 3). Both were perceived as structured approaches that enhanced the use of evidence while including contextual factors and facilitating consensus-building processes. Main barriers were lack of high-quality evidence for the effectiveness of PH interventions, limitations of the terminology or unclear boundaries between specific criteria, perceptions of missing criteria and the need for more guidance. Survey responses (n = 13) were consistent with these findings.

**Conclusion:**

Users of the two frameworks had an overall positive perception of the approaches, but several barriers remain. These experiences may change over time as the frameworks evolve. There is an evidence gap regarding users’ experience for other EtD frameworks.

## Introduction

Decision-making in public health (PH) aims to promote the health status of a group of people, from specific communities to entire continents [1,2]. It is a complex process that requires the perspective of a panel with diverse expertise profiles and the involvement of multiple stakeholders [3], including community members, health specialists, methodologists, economists and policymakers, among others.

Evidence-informed decision-making (EIDM) in PH should be based on the best available evidence, complemented by the expertise of panel members. It involves weighing the findings from effectiveness research but also from patients’ preferences, equity, acceptability and feasibility, among other aspects [4-7].

Evidence-to-decision (EtD) frameworks are structured approaches that aim to improve EIDM by explicitly considering critical criteria (i.e. any factor, domain or consideration relevant for decision-making), regardless of the type of decision (e.g. clinical recommendations, health systems or PH decisions) [8,9]. Previous reviews have mapped available frameworks for decisions about coverage [10] and environmental health interventions [11]. Since a positive experience with EtD frameworks among panellists is a major determinant for transparent and systematic EIDM [4], it is critical to clarify their current perspectives on the use of these frameworks. However, to our knowledge, no previous review has synthesised users’ experience with EtD frameworks for PH EIDM.

This is the second part of a broader scoping review. In the first publication, we reviewed the available EtD frameworks for PH EIDM, as well as examples focusing on infectious diseases [12,13]. This second manuscript summarises the experience of users of EtD frameworks for PH EIDM from the available literature and from direct contact with key stakeholders.

## Methods

For the first part of our project, we identified and summarised existing EtD frameworks for PH EIDM (research questions (RQ) 1 and RQ2) and identified examples of use in the field of infectious disease prevention and control (RQ3) [12,13]. Here we report the findings for users’ experiences with EtD frameworks for PH EIDM, as well as a description of facilitators and barriers for their implementation (RQ4). We aim to capture experiences across all PH EIDM fields (not necessarily limited to infectious diseases), as insights from other areas may also be valuable for infectious disease prevention and control. We followed the ‘Preferred Reporting Items for Systematic Reviews and Meta-Analysis’ (PRISMA) extension for scoping reviews to report our research [14]. The protocol for this research has been previously published [15].

### Eligibility criteria

For the broad scoping review, we included frameworks that describe any structured process (explicitly outlining domains, factors, or criteria considered) to support panels or users in transitioning from evidence to a recommendation or decision made on behalf of a population, that could potentially affect groups of people or entire populations [1,16]. For this second part of the project, we specifically considered documents describing users’ overall experience with the EtD frameworks identified in the broader review, as well as those identifying possible barriers and facilitators for their use. We excluded documents that did not use a structured framework, those using frameworks not intended for decision-making in a PH context (e.g. from an individual perspective) and documents in languages other than English.

Although the focus of our overall project was infectious disease prevention and control, here we consider any experience on the use of an EtD framework for PH decision-making, not limited to the infectious disease field. The rationale behind that decision is: (i) we expect limited literature assessing users’ experiences, and (ii) the experience from other areas is likely to also be useful for the infectious disease field.

### Information sources and search strategy

We searched MEDLINE and Health Systems Evidence (more information can be found in Supplement S1) up to December 2022, and hand searched publications from key organisations and scientific societies (details regarding the specific institutions, countries and websites searched are available in Supplement S2). For the latter, we used the following search terms on each organisation’s website: ‘handbooks’, ‘guidelines’, ‘recommendations’, ‘policy’, ‘decisions’, ‘HTA’ and ‘evidence’. We also tracked citations of all frameworks identified in the broader review using Google Scholar.

### Data management and selection process

After duplicates were removed, two authors (JB, YS) independently screened the references first by title and abstract and then by full text. We solved discrepancies by consensus, or by involving a third reviewer (DR, PA-C). One author (JB) hand searched websites and conducted the search, with a second reviewer (YS, DR, PA-C) cross-checking the results.

### Data collection and data synthesis

One author (JB) extracted first order (initial quotations) and second order (authors’ interpretations) constructs from the included documents to create third order (own) constructs. A second author (YS) cross-checked the constructs extracted. Then, one author (JB) generated descriptive themes of the users’ experiences with each framework, according to previously specified methods [17]. Two other authors (YS, PA-C) provided feedback and refined the thematic synthesis through discussion. We also interpreted and summarised the main experiences, facilitators and barriers in a tabular display.

To further complement these findings, we invited 58 key stakeholders selected from relevant institutions and organisations listed in Supplement S2, to complete a survey administered via EUSurvey (https://ec.europa.eu/eusurvey/). The survey consisted of 15 questions, primarily open-ended, focusing on the practical use of EtD frameworks within their institution, examples of use, adaptations, overall experience and perceived barriers and facilitators. Supplement S3 provides the details of the survey. Responses were qualitatively summarised and supported with verbatim quotes from respondents. To obtain deeper information on their experience, we invited five respondents for a semi-structured interview. We compared these findings with those from the literature.

## Results

From the initial 3,892 citations retrieved by the database search, we identified 42 eligible publications, of which nine described users’ experiences with EtD frameworks [18-26]. Additional sources provided 13 further references for the project, of which three [3,27,28] were deemed relevant. The Figure illustrates the selection process. Supplement S4 lists the studies excluded after full-text assessment and Supplement S5 includes the complete list of references used in the project.

**Figure:**
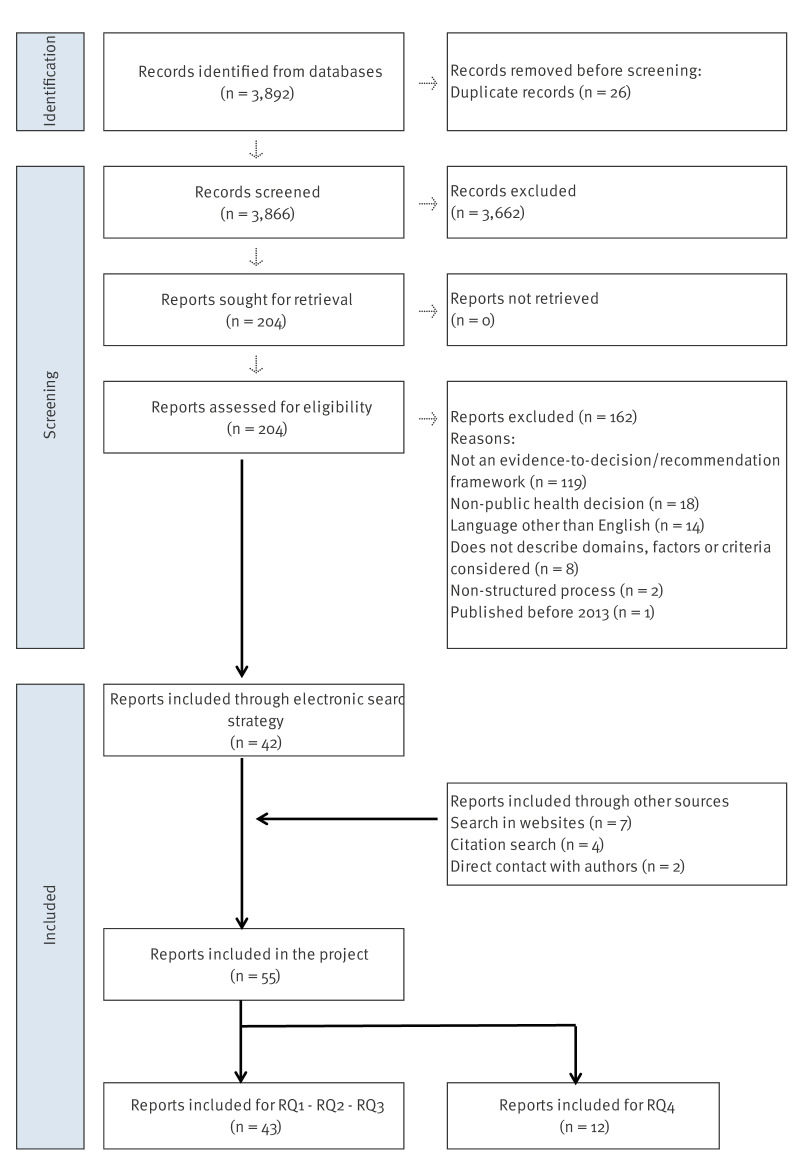
PRISMA flowchart for the selection process

RQ: Research question.

RQ1, RQ2 and RQ3 refer to the broader review, aiming to identify the existing frameworks (RQ1), the criteria considered within these frameworks (RQ2) and examples for PH EIDM in infectious diseases (RQ3). This review focuses on the experience of users of the frameworks (RQ4).

### Literature findings

We included 12 studies reporting users’ experiences. Nine of these studies explored experiences with the ‘Grading of Recommendations Assessment, Development and Evaluation’ (GRADE) EtD framework [18-20,22-24,27,28], and three with the World Health Organization (WHO) ‘INTEGRATe Evidence’ (INTEGRATE) EtD framework [25,26,29]. No documented experiences were found for the other 13 frameworks identified the first stage of the project. Studies on experiences with using the GRADE EtD framework were published between 2016 and 2022 [18-20,22-24,27,28]. Six studies included the perspective of stakeholders and people involved in guideline development [18-23]. One of these studies was a literature review [28], one study combined a literature review with interviews of guideline developers [27] and one study simulated a guideline panel to apply the framework [24]. Studies reporting experiences with using the WHO-INTEGRATE framework were published between 2022 and 2023 and included perspectives from individuals with experience in guideline development and evidence-based policy [3,25,26]. Table 1 presents the main characteristics of these studies.

**Table 1:** Main characteristics of the studies describing experiences of using evidence-to-decision frameworks^a^

Study	Framework assessed	Aim of the study	Overall methods	Participants
Guldbrandsson 2016 [20]	GRADE EtD framework	To assess the applicability of the framework as a tool for dissemination and implementation of recommendations in the PH field in Sweden.	The framework was presented and discussed in interviews with stakeholders and governmental organisations and tested in panels. Authors performed content analysis.	Stakeholders from local, regional and national levels in the PH field.
Neumann 2016 [21]	GRADE EtD framework	To report on the first experience with the EtD framework for clinical recommendations in real guideline panels.	Authors requested feedback from methodologists supervising panels that developed 15 international guidelines. Pre-specified domains to code the information.	Ten methodologists leading guidelines on a variety of topics. All participants had postgraduate training in health research methods or a related discipline, and all were members of the GRADE working group.
Li 2018 [19]	GRADE EtD framework	To explore how panellists adhered to GRADE criteria and sought to identify any emerging non-GRADE criteria when using the EtD framework.	Conventional and summative qualitative analysis to identify themes emerging from face-to-face, panel meeting discussions. Forty-eight members from 12 countries participated in developing five guidelines for the management of venous thromboembolism by the American Society of Haematology.	The decision-making panels consisted of 48 members (40 content experts and methodologists, eight patient representatives) from multiple countries (mostly North America and Europe).
Rosenbaum 2018 [18]	GRADE EtD framework	To help decisionmakers achieve fairness in their decision-making by creating tools that would facilitate three process elements.	Broad range of structured, semi-structured and open-ended methods to inform cycles of prototyping and feedback: piloting in actual guideline projects, participatory and non-participatory observation of guideline panels and workshops, prototype sketching, testing examples, user-test interviews, stakeholder feedback, questionnaires, surveys and discussion in face-to-face meetings. Iterative process for adjustments and improvements. No formal qualitative analysis.	People in organisations involved in decision-making and dissemination (e.g. guideline producers, panel members) and people who would use this information (e.g. policymakers, health professionals, the public).
Meneses-Echavez 2021 [22]	GRADE EtD framework	To describe users’ experiences with the interactive evidence-to-decision (iEtD) framework, identifying main barriers and facilitators related to its use.	Semi-structured interviews with iEtD registered users. Honeycomb framework used to guide the interviews and explore users’ experiences with the iEtD [40]. Content analysis.	Eight methodologists registered in the iEtD database from national or international organisations that developed guidelines.
Stalteri Mastrangelo 2021 [28]	GRADE EtD framework	To analyse the consideration of antimicrobial resistance within tuberculosis, gonorrhoea and respiratory tract infections, and for guidelines considering antimicrobial resistance, their quality and potential for contextualisation.	Systematic review of clinical guidelines.	Not applicable.
Friesen 2022 [23]	GRADE EtD framework	To demonstrate how the GRADE EtD framework for health systems and PH decisions can be applied to formulate recommendations and make decisions in national food fortification programming.	Description of the experience of applying the GRADE EtD framework to a food fortification programme.	Authors and a small group of stakeholders from governmental organisations involved in Nigeria’s national food fortification programme.
Moleman 2022 [27]	GRADE EtD framework	To examine guideline quality in relation to the availability of certain types of evidence and to reflect on the implications of CPGs' promise to improve the quality of care practices.	Mixed‐methods study consisting of two phases: a quantitative evaluation of 62 Dutch clinical practice guidelines using AGREE tool, and qualitative follow‐up interviews about experiences with the development process.	Thirteen guideline developers of the assessed guideline and six other experts in national and international guideline development.
Stadelmaier 2022 [24]	GRADE EtD framework	To illustrate the application of GRADE EtD frameworks in the process of nutrition-related policy-making for a European country.	Illustration of the process of moving from evidence to recommendations by applying the EtD frameworks to a fictitious example. Sugar-sweetened beverage taxation based on energy density was chosen as an example application.	Authors created a fictitious guideline panel.
Murano 2022 [26]	WHO-INTEGRATE	To describe the methods used to apply WHO-INTEGRATE. It also summarises the results of an evidence review for each of the EtD criteria applied to three prioritised labour induction topics. To reflect on the methods, process and evidence review outputs. It discusses limitations and challenges, gaps in the evidence base and opportunities to improve the process of applying WHO-INTEGRATE.	Adoption of WHO-INTEGRATE framework to consider key criteria and subcriteria relevant to the intervention. Searches for qualitative, cost and cost-effectiveness studies, and other types of evidence. Iterative approach for interpreting the evidence. Summary of the findings for decisionmakers, and reflection about the process.	Two researchers (social science and PH background), with experience in evidence synthesis and evidence-based policy, from Australia.
Stratil 2022 [25]	WHO-INTEGRATE	To assess WHO-INTEGRATE framework comprehensiveness and usefulness for PH and health policy decision-making.	Qualitative study, comprising interviews and focus group discussions. Qualitative content analysis.	Nine experts involved in WHO guideline development and 40 health decisionmakers from Brazil, Germany, Nepal and Uganda (including infectious diseases as thematic area).
Wabnitz 2023 [3]	WHO-INTEGRATE	To identify lessons learnt about strengths and weaknesses of the guideline development process as perceived by the different groups involved, in the context of a guideline development process about measures for the prevention and control of SARS-CoV-2 transmission in schools.	Semi-structured interviews. Deductive-inductive thematic qualitative text analysis according to Kuckartz [41], structuring findings using a category system.	Fifteen persons involved in guideline development, including the following: four members of the guideline secretariat, four scientists, four members of the school family, two PH practitioners and one observer.

AGREE: Appraisal of Guidelines, Research and Evaluation; CPG: Clinical practice guideline; EtD: evidence-to-decision; GRADE: Grading of Recommendations Assessment, Development and Evaluation; iEtD: interactive evidence-to-decision; PH: public health; SARS-CoV-2: severe acute respiratory syndrome coronavirus 2; WHO: World Health Organization; WHO-INTEGRATE: World Health Organization INTEGRATe Evidence.

^a^ Although all the studies listed here describe users’ experience with an EtD framework for PH decision-making, they are not necessarily specific for the infectious disease field.

The main facilitators identified for GRADE EtD framework use were stakeholder engagement; the possibility of tailoring the framework; and the provision of clear guidance and previous training. Barriers were related to the unavailability of high-quality evidence (as considered by GRADE) for PH; the need for prior skills and knowledge; the misunderstanding of the wording for some criteria; the presence of domineering participants in the panels; and time constraints.

Among the facilitators for using the WHO-INTEGRATE framework, the literature described the provision of previously summarised evidence and the availability of diverse expertise through the formation of multidisciplinary panels. The main barriers were lack of guidance; resource constraints; the limited availability of evidence; and the common misunderstanding of the boundaries between different criteria and subcriteria. Table 2 summarises and Supplement S6 details the main experiences, facilitators and barriers identified by the literature.

**Table 2:** Summary of the main facilitators and barriers for the use and implementation of the identified evidence-to-decision frameworks

Overall experience	Barriers	Facilitators
**GRADE EtD framework**		
This framework helps reach consensus among panel members. Panel discussion allows perspectives’ adjustment and reach consensus [20]. GRADE EtD increases the systematic use of evidence, raises awareness of local evidence gaps [23]. GRADE EtD criteria contributes to most of the discussions within the included panels [19]. GRADE enhances consideration of contextual factors [28]. GRADE is perceived as a methodological improvement compared with other approaches, more transparent and systematic [27]. EtD provides structure and facilitates management of the panel [18]. EtD as structured, facilitating discussions and decision-making [24].	Complicated evidence grading system [18,20,22,27]. Lacking evidence or inappropriateness of RCT as for PH interventions [19,20,27]. Difficulties for decision-making when multiple comparisons are available [27]. Specific challenges for the chair such as time, domineering participants, biases in discussion and when introducing information [18]. Specific barriers for each factor, such as troubles assessing magnitude of effect for ‘balance of benefits and harms’, mixed views about ‘resource use’ and some redundancy and overlap among criteria [27]. Lack of implementation/adaptation considerations [20]. Missing factors to be addressed, including political and social circumstances [19,23] or some factors not relevant for specific circumstances [27]. Suboptimal wording [18,27]. More guidance is needed for terms such as ‘values’ [18,27], ‘equity’ [22,27], ‘acceptability’ [27].	Stakeholder engagement [18,20]. Provision of guidance and training [19,20,22]. Tailoring of factors according to context [18,20,22]. Facilitators for specific factors: (i) affordability considerations at different levels and cost-effectiveness analyses as key topics for assessing resources and costs [19], (ii) contextualisation and discussing desirable and undesirable effects facilitate balance of benefits and harms [19], (iii) patients’ values and preferences addressed by patient representatives and supplemented by clinical expertise and experience of patients’ values [19]. Facilitators for Panel discussion and decision-making: sufficient evidence [19]; clinical experience [19]; good chair [18]; structure of the framework including ‘research priorities’ [27], ‘additional considerations’ and ‘conclusions’ sections [18,22].
**WHO-INTEGRATE**		
WHO-INTEGRATE allows the exploration of health rights and inequity in a detailed, systematic and transparent way, especially in absence of conclusive evidence [3,26]. WHO-INTEGRATE framework separates individual and population perspectives, range of feasibility considerations, broad perspective beyond health implications [25]. WHO-INTEGRATE framework allowed health and societal implications to be considered systematically, mostly informed by anecdotal expertise due to lack of studies and of professional expertise [25]. Participants appreciate transparent, democratic and anonymous consensus-building procedures [3].	Process may be overwhelming due to its complexity and workload [3]. Limited availability of evidence and lack of high-quality evidence [3,25]. Context-dependence may limit applicability [25]. Specific barriers for each factor, mainly related to hierarchy of the questions, overlap among criteria and subcriteria, and blurry boundaries [25]. Challenges related to the decision-making process, such as balancing different perspectives, selecting which type of evidence to consider, weighting criteria, prioritising endpoints for outcomes, dealing with conflicts of interest and managing hierarchy among panel members [3]. Possible missing aspects, such as intervention sustainability, reliability and quality of an intervention, outcomes related to well-being, political feasibility [25]. More guidance is needed regarding the assessment of some specific criteria or subcriteria [25], and the process of selecting panel members [3].	Stakeholder engagement [25]. Enhancement of WHO-INTEGRATE use by systematic mapping methods, consideration of other frameworks, complementary and previous identification and appraisal of scientific literature [3,26]. Tailoring of the framework, in terms of planning resource and time allocation [3]. Facilitators for specific factors: (i) trial-based studies may inform resources use [26], (ii) considering population characteristics and settings at an early stage may enhance the assessment of equity considerations [26], (iii) integrating disciplines such as social sciences may improve discussions about equity [26]. Facilitators for Panel discussion and decision-making: team collaboration [26]; provision of multi-layered evidence presentation format [26]; considering evidence alongside with different types of panel members’ expertise (scientific expertise, practical expertise, lived experience) [3].

EtD: evidence-to-decision; GRADE: Grading of Recommendations Assessment, Development and Evaluation; PH: public health; RCT: randomised controlled trials; WHO-INTEGRATE: World Health Organization INTEGRATe Evidence.

### Experience using the Grading of Recommendations Assessment, Development and Evaluation evidence-to-decision framework

#### Overall perception of the framework

Six studies explored the overall experience of using the GRADE EtD framework, all reporting an overall positive perception of the process [18,20-22,24,27]. Supplement S7 provides the detailed summary of the literature findings of the users’ experiences with the GRADE EtD framework. The framework was perceived as a structured, comprehensive and transparent approach that increased the systematic use of evidence, facilitating discussion, decision-making and consideration of contextual factors [28].

#### Panel composition and workflow

Users perceived the panel composition as critical, highlighting the importance of reflecting all interests related to the final decisions [20]. Having a trained chair who could deal with time constraints, domineering participants and bias in the panel discussions was perceived a key factor for a successful use of the framework [18]. The workflow among panels using the GRADE EtD was variable [18].

#### Assessing the evidence in the public health field

Users noted that randomised controlled trials are scarce and may not always be the most appropriate research design in the PH field [20,27]. Other studies (not specifically assessing the experience of using the framework itself) have also highlighted similar problems [30,31].

#### Experience using the framework criteria

Decision-making processes took longer if no evidence or only low-quality evidence was available [19]. Users pointed out specific issues for some criteria, such as confusion related to the terms ‘values’ and ‘equity’ [18,21,22], and had mixed views about the ‘resource use’ criterion depending on the inclusion of health economists among panels [21]. Users perceived overlap between some criteria (specifically between ‘acceptability’ and ‘feasibility’, and also between ‘values and preferences’ and ‘resource consideration’) [21,22].

#### The value of tailoring the framework

Users valued the option of tailoring the framework in different ways, as sometimes the framework may contain sections not relevant for specific circumstances [18,20-23]. Criteria for the GRADE EtD framework may not directly address all the relevant factors for specific decision-making processes, which may lead to the need to include new considerations [20,22,23], such as political or social factors [23], individual autonomy and method sustainability [20] or legal considerations [19].

#### The need for training

Using the GRADE EtD framework requires prior training in overall GRADE methodology or in using specific tools (e.g. the interactive web-based evidence-to-decision tool iEtD) [21,22]. Panel discussions were mainly driven by the predefined criteria, which may be due to the comprehensiveness of the criteria, or a bias from prior training [19].

#### Importance of language and wording

Some users stated that the wording of terminology and signalling questions from the GRADE EtD framework in the assessment section was unclear [22]. The wording of recommendations represented an important part of the panel discussions [18,19].

#### Experience with the interactive web-based evidence-to-decision tool

The overall experience using an iEtD was positive, with users describing the tool as intuitive, simple, easy-to-use, well-organised and freely available [18,22]. Users appreciated the help sections, the distinction between evidence and judgements/additional considerations and the online voting option [18,22]. However, additional workload was also reported when working with large groups or large amounts of evidence [22] and some reported a burden related to the process of learning a new technology [18].

### Experience using the World Health Organization INTEGRATe Evidence evidence-to-decision framework

#### Overall perception of the framework

Three studies including 26 participants described the overall experience of using the WHO-INTEGRATE framework as positive [3,25,26]. Supplement S8 provides the detailed summary of the literature findings of the users’ experiences with the WHO-INTEGRATE framework. Overall, the framework was perceived as useful, detailed, structured, systematic and transparent, allowing the separation of different perspectives and consideration of feasibility and broader implications beyond health [3,25,26]. However, some considered the framework to be too comprehensive and questioned its added value [3,25]. Users perceived that more guidance is still needed [25].

#### Panel members’ profiles, roles and hierarchy

Collaboration among people with different profiles in the developing team was positively perceived, enhancing consistency, legitimacy and interpretation of findings [3,26].

Three types of expertise were described: (i) scientific expertise, grounded in scientific studies and disciplinary knowledge, (ii) practical expertise, derived from implementing the practical measures, and (iii) lived experiences of those affected by the measures [3]. Profiles influenced differential insights into how to consider or interpret different types of evidence and outcomes [3].

Most users perceived a hierarchy among panel members that influenced the discussions, determined by member profile, seniority, academic credentials, professional experience, institution and eloquence [3].

#### The concept and consideration of evidence

Authors reflected that the use of an iterative framework may facilitate the extraction of most relevant findings and that three outputs should be provided to the panel: (i) a high-level summary of evidence for each criterion, (ii) a supplemental file with detailed findings of evidence, and (iii) an evidence gap map for each criterion [26]. Participants agreed that evidence was critical for the guideline development process, but users with different profiles reported mixed views about how to consider different types of evidence, emphasising the need to develop a shared understanding of the concept and role of evidence [3].

#### Experience using the framework criteria

Overall, all WHO-INTEGRATE criteria and subcriteria were seen as important for real-world PH EIDM, and users thought that none should be dropped [25]. However, due to the complexity and additional workload of actually using the framework, some users reported skipping (or prioritising) specific domains, which may diminish the value of the final product [3,25].

Participants referred to issues with wording and definitions (specifically in the ‘equity, equality and non-discrimination’ and ‘societal implications’ criteria), and overlap, redundancy or delineation problems for several criteria and subcriteria, as well as missing aspects (specifically for the ‘balance of benefits and harms’, ‘human rights and socio-cultural acceptability’, and ‘equity, equality and non-discrimination’ criteria) [25]. However, WHO-INTEGRATE developers stated that the criteria identified as missing were in fact covered by the framework [25].

Focus groups that used the framework felt that it successfully encompassed their reasoning through the discussion of all criteria, despite not always addressing each specific subcriterion [3,25]. Some considered that all the new subcriteria provided by WHO-INTEGRATE could also be addressed within the GRADE EtD framework [25].

#### Perspectives on the development process

Users described the identification of evidence for specific domains as challenging, in which case lived experience was considered important [3,25]. However, balancing different perspectives within the panel was challenging [3]. Many aspects of the WHO-INTEGRATE framework were noted as context-dependent, which could limit the applicability of the recommendations for global guidelines [25].

The iterative process of working in small groups plus subsequent full-panel consensus was efficient and goal-oriented, but time pressure and lack of resources may create a burden due to excessive task assignments and may also hinder in-depth discussion of the recommendations [3].

### Survey findings

We obtained 13 responses to our survey. Six participants were from European countries (Belgium, Croatia, Estonia, France, Norway and the Netherlands) and seven from two non-European high-income countries (three from Australia and four from Canada). Seven respondents reported using the GRADE EtD framework, with no responses regarding any other framework. The GRADE EtD framework was perceived as structured and well accepted. However, some concerns were raised regarding the appropriateness of the framework for the PH field. The lack of evidence was considered an important challenge, as well as the difficulty of using this framework when rapid decision-making is needed. In addition, respondents highlighted the need for specialised knowledge as a potential barrier. Supplement S9 provides a detailed summary of the survey responses. None of the five participants invited to a semi-structured interview were available.

## Discussion

We assessed 12 studies reporting users’ experience with only two of the 15 previously identified EtD frameworks for PH EIDM - the GRADE EtD framework (nine studies) and the WHO-INTEGRATE framework (three studies). Overall, both frameworks were perceived as structured approaches that enhanced EIDM by facilitating the systematic use of evidence, the consideration of contextual factors and consensus-building processes. In both cases, the main barriers were related to the lack of high-quality evidence in the PH field, problems with the wording of or boundaries around specific criteria, perceptions of missing criteria and the need for more guidance. Users of the GRADE EtD framework also reported issues with the decision-making process itself (e.g. managing multiple comparisons, handling large groups, specific challenges for the chair), barriers for specific domains (such as ‘resource use’ and overlapping assessments of different criteria) and insufficient implementation considerations of the framework. On the other hand, WHO-INTEGRATE was sometimes perceived as an overwhelming process to adopt, with limited applicability in some contexts, along with criticism about boundaries and limits for several criteria and subcriteria and issues with the decision-making process itself.

To the best of our knowledge, this is the first evidence synthesis on users’ experiences with EtD frameworks for EIDM in PH. Although some studies have documented users’ experiences with GRADE evidence tables [32] and tools for completing EtD frameworks [22], overall users’ experiences with EtD frameworks for PH EIDM have received less attention.

Some studies have explored the experiences of PH EIDM with no explicit use of an EtD framework [33,34]. These studies describe some challenges that are consistent with our findings (such as difficulties related to the availability of evidence), but highlighted others that were contradictory, such as lack of transparency, clarity and structure of the process [33,34]. Therefore, compared with these experiences, the use of EtD frameworks for EIDM may have some important advantages.

This study has some potential limitations. We only searched two electronic databases up to December 2022. However, we conducted complementary search strategies (i.e. hand searched several key organisations’ websites and tracked citations) that allowed us to identify additional studies relevant to our research question. Since our focus was exploratory, we did not appraise the methodological quality of the included studies [35]. Despite sending more than 50 invitations for our survey, we received only 13 responses, with no successful responses for the planned interviews. Our findings may be prone to nonresponse bias, with those people not responding having experienced a less satisfactory use of an EtD framework, or not being users of these frameworks at all. Nevertheless, since the responses were mostly consistent with the existing literature, this bias is unlikely to be particularly relevant for our findings. While most of the user experiences in our review did not focus specifically on PH EIDM for infectious diseases, their applicability to this field of research is plausible. The use of duplicate and independent approaches for study identification and data extraction strengthened data accuracy in this review.

The users’ experiences mapped out in this scoping review along with the feedback provided by the survey responses may inform either the development of new EtD frameworks or improvements of the existing frameworks. For example, since randomised controlled trials are infrequent in PH, our findings may encourage the development of improved guidance for making recommendations in the presence of low or very low quality evidence derived from other study designs. In addition, clearer wording and training should also be considered as part of strategies to improve users’ experiences with EtD frameworks. Guidance documents for guideline development should include these strategies to facilitate a balanced experience of using the EtD frameworks and their related tools (e.g. GRADE iEtD).

Our findings on the WHO-INTEGRATE EtD framework, launched in 2018 [36], may seem scarce compared with the more widely studied GRADE EtD framework, whose first publication in a peer-reviewed journal was in 2016 [8]. However, it should be noticed that the GRADE working group has been constantly developing, disseminating and implementing its methods for over 20 years, which may be, at least partially, the reason why it is currently the most used EtD framework worldwide [9]. Today, both WHO-INTEGRATE and GRADE EtD frameworks are backed by active working groups behind their development [25,37]. Therefore, the interpretation of our findings should remain dynamic, evolving in parallel with the ongoing development and refinements of these frameworks.

### Reflexivity statement

Reflexivity is a crucial step within qualitative research, which implies self-reflection on how the researchers’ subjectivities and biases shape the research process [38,39]. The project team was composed of researchers currently based in Europe. However, two authors (JB, JM-E) are from South America, and one author (YS) is from Asia, and they all maintain active professional connections with their countries of origin. Therefore, as a research group, we believe we have a broad and diverse perspective when interpretating our findings. Through collaborative work on other projects, and considering the survey respondents, we believe other contexts such as North America or Oceania may be well represented, although we acknowledge we may have incurred in a potential bias due to an underrepresentation of other geographical areas.

All authors have experience in EIDM and in using EtD frameworks with guideline panels. One author (PAC) was involved in the development of the GRADE EtD framework and four authors are active members of the GRADE working group. While experience in the field is an advantage, we acknowledge that these relationships may have influenced our interpretations of the users’ experiences related to the GRADE EtD framework. However, we did not limit our study selection to any specific framework. We cross-checked and discussed all our findings and conducted data analysis using validated methods [17]. It is important to highlight that the development of the WHO-INTEGRATE EtD framework was partially based on the GRADE EtD framework [36], and, in consequence, it is possible that our experience with the GRADE approach may have also influenced the findings for the WHO-INTEGRATE framework.

## Data Availability

All data are available in the Supplementary material.

## Supplementary Data

820000 Supplementary Material
