# Supplementary material for: Users’ experience of frameworks to support evidence-informed decision-making in public health: a scoping review
Source: Euro Surveill. 2025 May 15;30(19):2400184. doi: 10.2807/1560-7917.ES.2025.30.19.2400184 (PMC12083067; doi:10.2807/1560-7917.ES.2025.30.19.2400184)
Supplement: Supplementary Material [file 24-00184_CARVALHO_GOMES_Supplement.pdf]

This supplementary material is hosted by *Eurosurveillance* as supporting information alongside the article ‘Users’ experience of frameworks to support evidence-informed decisión-making in public health: a scoping review’, on behalf of the authors, who remain responsible for the accuracy and appropriateness of the content. The same standards for ethics, copyright, attributions and permissions as for the article apply. Supplements are not edited by *Eurosurveillance* and the journal is not responsible for the maintenance of any links or email addresses provided therein.

Supplement S1. Search strategy for MEDLINE/PubMed and Health Systems Evidence

**MEDLINE/PubMed**<br><br>***Date of last search: December 19, 2022***	
#1	"Decision Making"[Majr]
#2	evidence[ti]
#3	decision*[ti]
#4	recommendation*[ti]
#5	policy[ti]
#6	priority[ti]
#7	priorities[ti]
#8	prioritisation[ti]
#9	prioritization[ti]
#10	#1 OR #2 OR #3 OR #4 OR #5 OR #6 OR #7 OR #8 OR #9
#11	"Evidence-Based Medicine"[Mesh]
#12	"Health Policy"[Mesh]
#13	#11 OR #12
#14	approach*[tiab]

#15	formulat*[tiab]
#16	develop*[tiab]
#17	review*[tiab]
#18	synthes*[tiab]
#19	synthez*[tiab]
#20	guidance[tiab]
#21	criteria[tiab]
#22	methodolog*[tiab]
#23	inform*[tiab]
#24	guideline*[tiab]
#25	#13 OR #14 OR #15 OR #16 OR #17 OR #18 OR #19 OR #20 OR #21 OR #22 OR #23 OR #24
#26	framework*[ti]
#27	#10 AND #25 AND #26
#28	approach*[ti]
#29	formulat*[ti]
#30	develop*[ti]
#31	review*[ti]
#32	synthes*[ti]
#33	guideline*[ti]
#34	guidance[ti]

#35	criteria[ti]
#36	methodolog*[ti]
#37	inform*[ti]
#38	#13 OR #27 OR #28 OR #29 OR#30 OR #31 OR #32 OR #33 OR #34 OR #35 OR #36 OR #37
#39	theor*[ti]
#40	#10 AND #38 AND #39
#41	#27 OR #40

**Health Systems Evidence**<br>***Date of last search: December 19, 2022***
(evidence OR decision* OR recommendation* OR policy OR priority OR priorities OR prioritisation OR prioritization) AND (approach* OR formulat* OR develop* OR review* OR synthes* OR synthez* OR guidance OR criteria OR methodolog* OR inform* OR guideline* OR tool OR tools) AND (framework* OR theor*)

Supplement S2. List of Institutions and Organizations for Web Search.

Institution or organisation	Country	Website
Federal Ministry of Health	Austria	http://www.bmg.gv.at/
Sciensano	Belgium	https://www.sciensano.be/en
National Centre of Infectious and Parasitic Diseases	Bulgaria	http://www.ncipd.org/
Croatian National Institute of Public Health	Croatia	http://www.hzjz.hr/epocetna.htm
Directorate of Medical and Public Health Services	Cyprus	http://www.moh.gov.cy/moh/moh.nsf/index_en/index_en
National Institute of Public Health (NIPH)	Czech Republic	http://www.szu.cz
Danish Health and Medicines Authority	Denmark	http://www.sundhedsstyrelsen.dk/English.aspx
Health Board	Estonia	http://www.terviseamet.ee
Finnish Institute for Health and Welfare	Finland	http://www.thl.fi
French Public Health Agency	France	http://www.santepubliquefrance.fr
Robert Koch Institute	Germany	http://www.rki.de
National Public Health Organization	Greece	https://eody.gov.gr/eody/
National Public Health Center, Ministry of Human Capacities	Hungary	https://2015-2019.kormany.hu/en/ministry-of-human-resources
Centre for Health Security and Communicable Disease Control, Directorate of Health	Iceland	http://www.landlaeknir.is/
Health Protection Surveillance Centre	Ireland	https://www.hpsc.ie/
Ministry of Health	Italy	http://www.salute.gov.it/
Centre for Disease Prevention and Control	Latvia	http://spkc.gov.lv/
Principality of Liechtenstein	Liechtenstein	http://www.ag.llv.li
Ministry of Health	Lithuania	http://www.sam.lt/
Health Directorate	Luxembourg	https://sante.public.lu/fr.html
Superintendence of Public Health	Malta	https://deputyprimeminister.gov.mt/en/sph/Pages/Superintendence-of-Public-Health.aspx

Classified as ECDC NORMAL

National Institute for Public Health and the Environment (RIVM)	Netherlands	http://www.rivm.nl/
Norwegian Institute of Public Health	Norway	http://www.fhi.no/
National Institute of Public Health/National Institute of Hygiene	Poland	http://www.pzh.gov.pl/
Directorate General of Health	Portugal	http://www.dgs.pt/
National Institute of Public Health	Romania	http://www.insp.gov.ro/
Public Health Authority of the Slovak Republic	Slovakia	http://www.uvzsr.sk/en/
National Institute of Public Health (NIJZ)	Slovenia	http://www.nijz.si
Ministry of Health, Social Services and Equality	Spain	http://www.msssi.es
Public Health Agency of Sweden	Sweden	https://www.folkhalsomyndigheten.se/
Africa Centres for Disease Control and Prevention (Africa CDC)	Africa	https://africacdc.org/
Australian Government Department of Health and Aged Care	Australia	https://www.health.gov.au/
Australian Commission on Safety and Quality in Health Care	Australia	https://www.safetyandquality.gov.au/
Ministério da Saúde Brazil	Brazil	https://www.gov.br/saude/pt-br
Public Health Agency of Canada	Canada	https://www.canada.ca/en/public-health.html
Canadian Task Force on Preventive Health Care	Canada	https://canadiantaskforce.ca/
Caribbean Public Health Agency	Caribbean	https://carpha.org/
Chinese Center for Disease Control and Prevention	China	https://www.chinacdc.cn/en/
Israel Ministry of Health	Israel	https://www.gov.il/en/departments/ministry_of_health/govil-landing-page
Japanese Ministry of Health, Labour and Welfare	Japan	https://www.mhlw.go.jp/english/
Korea Disease Control and Prevention Agency	Korea	https://www.kdca.go.kr/index.es?sid=a2
Mexico Ministry of Health	Mexico	https://www.gob.mx/salud
Singapore Ministry of Health	Singapore	https://www.moh.gov.sg/
Thailand Ministry of Health	Thailand	https://p4h.world/en/member/ministry-public-health-thailand
UK Health Security Agency	UK	https://www.gov.uk/government/organisations/uk-health-security-

		agency
Center for Disease Control and Prevention	USA	https://www.cdc.gov/
World Health Organization	International	https://www.who.int/
National Institute for Health and Care Excellence (NICE)	UK	https://www.nice.org.uk/
Health Information and Quality Authority	Ireland	https://www.hiqa.ie/
National Collaborating Centre for Methods and Tools	Canada	https://www.nccmt.ca/
Cochrane Public Health	International	https://ph.cochrane.org/
GRADE Working Group (public health group)	International	https://www.gradeworkinggroup.org/
Joanna Briggs Institute	International	https://jbi.global/
The Community Guide	-	https://www.thecommunityguide.org/
Joint Research Commission (JRC)	International	https://joint-research-centre.ec.europa.eu/index_en
European Commission	International (Europe)	https://commission.europa.eu/index_en
European Union (centres and agencies related to health)	International (Europe)	https://european-union.europa.eu/
International Network for Government Science Advice	-	https://ingsa.org/
Guideline International Network (GIN)	International	https://g-i-n.net/get-involved/resources
Infectious Disease Society of America (IDSA)	International	https://www.idsociety.org/

Supplement S3. Institutions contacted and Survey Distributed to Key Institutions

3.1 List of institutions contacted

Institution	Email address or link	Date sent
Federal Ministry of Health, Austria	post@sozialministerium.at	19-04-23
Sciensano, Belgium	info@sciensano.be	19-04-23
Croatian National Institute of Public Health	ravnateljstvo@hzjz.hr	19-04-23
Directorate of Medical and Public Health Services, Cyprus	perm.sec@moh.gov.cy	19-04-23
National Institute of Public Health (NIPH), Czech Republic	zdradust@szu.cz	19-04-23
Danish Health and Medicines Authority	sst@sst.dk	19-04-23
Health Board, Estonia	info@terviseamet.ee	19-04-23
Finnish Institute for Health and Welfare	mika.salminen@thl.fi	19-04-23
Robert Koch Institute, Germany	https://www.rki.de/EN/Content/Institute/Locations/Contact/zentrale_node.html	(link broken)
National Public Health Organization, Greece	info@eody.gov.gr	19-04-23
National Public Health Center, Ministry of Human Capacities, Hungary	ugyfelszolgalat@emmi.gov.hu	19-04-23
Centre for Health Security and Communicable Disease Control, Directorate of Health, Iceland	mottaka@landlaeknir.is	19-04-23
Health Protection Surveillance Centre, Ireland	info@hpsc.ie	19-04-23
Ministry of Health, Italy	segretariato.generale@sanita.it	19-04-23
Centre for Disease Prevention and Control, Latvia	pasts@spkc.gov.lv	19-04-23
Principality of Liechtenstein	info.ag@llv.li	19-04-23
Ministry of Health, Lithuania	ministerija@sam.lt	19-04-23
Health Directorate, Luxembourg	https://sante.public.lu/fr/support/contact.html	19-04-23
Superintendence of Public Health, Malta	sph.health@gov.mt	19-04-23
National Institute for Public Health and the Environment (RIVM), Netherlands	info@rivm.nl	19-04-23
Norwegian Institute of Public Health, Norway	trygve.ottersen@fhi.no	19-04-23
National Institute of Public Health/National Institute of Hygiene, Poland	pzh@pzh.gov.pl	19-04-23
Directorate General of Health, Portugal	geral@dgs.min-saude.pt	19-04-23

Public Health Authority of the Slovak Republic	uvzsr@uvzsr.sk podatelna@uvzsr.sk	19-04-23
National Institute of Public Health (NIJZ), Slovenia	info@nijz.si	19-04-23
Ministry of Health, Social Services and Equality, Spain	oiac@sanidad.gob.es	19-04-23
Public Health Agency of Sweden	info@folkhalsomyndigheten.se	19-04-23
Africa Centres for Disease Control and Prevention (Africa CDC)	africacdc@africa-union.org	19-04-23
Australian Government Department of Health and Aged Care	CDNA.SoNG@health.gov.au	19-04-23
Australian Commission on Safety and Quality in Health Care	mail@safetyandquality.gov.au	19-04-23
Public Health Agency of Canada	cphocorrespondence@phac-aspc.gc.ca	19-04-23
Canadian Task Force on Preventive Health Care	https://canadiantaskforce.ca/contact/	19-04-23
Caribbean Public Health Agency	postmaster@carpha.org	19-04-23
Israel Ministry of Health	Call.Habriut@moh.health.gov.il	19-04-23
Japanese Ministry of Health, Labour and Welfare	www-admin@mhlw.go.jp	19-04-23
Mexico Ministry of Health	informes_cenetec@salud.gob.mx	19-04-23
Singapore Ministry of Health	NIPC_Sec@moh.gov.sg	19-04-23
P4H	meyerc@who.int tomassinie@who.int	19-04-23
UK Health Security Agency	enquiries@ukhsa.gov.uk	19-04-23
Center for Disease Control and Prevention, USA	https://wwwn.cdc.gov/dcs/contactus/form	19-04-23
World Health Organization	https://www.who.int/about/contact-us	(no email nor form to contact)
National Institute for Health and Care Excellence (NICE), UK	nice@nice.org.uk	19-04-23
Health Information and Quality Authority, Ireland	info@hiqa.ie	19-04-23
National Collaborating Centre for Methods and Tools, Canada	nccmt@mcmaster.ca	19-04-23
Cochrane Public Health	CPHE@cochrane.at	19-04-23
GRADE Working Group (public health group)	mail@gradeworkinggroup.org	19-04-23
Joanna Briggs Institute	jbi@adelaide.edu.au	19-04-23
The Community Guide	communityguide@cdc.gov	19-04-23
International Network for Government Science Advice	info@ingsa.org	19-04-23
Guideline International Network (GIN)	chair@g-i-n.net	19-04-23
Infectious Disease Society of America (IDSA)	https://www.idsociety.org/contact-us/	19-04-23
Czech National Centre for Evidence-Based Healthcare and Knowledge	klugar@med.muni.cz	27-04-23

Public Health Agency, Sweden	par.vikstrom@folkhalsomyndigheten.se	27-04-23
Australian Commission on Safety and Quality in Health Care	alice.bhasale@safetyandquality.gov.au	27-04-23
Australian Commission on Safety and Quality in Health Care	Davina.Ghersi@nhmrc.gov.au tari.turner@monash.edu	27-04-23
National Institute for Health and Care Excellence (NICE), UK	Kate.Kelley@nice.org.uk	09-05-23
Service évaluation en santé publique et évaluation des vaccins, France	p.minayaflores@has-sante.fr	09-05-23
Federal Ministry of Health, Austria	christina.dietscher@bmg.gv.at	09-05-23
Dirección General de Salud Pública (DGSP), Spain	dgsp@sanidad.gob.es	11-05-23

**Institutions listed in appendix 2 but not here were not contacted for the survey due to lack of contact information available on the webpage.*

3.2 Content of the survey

Question	Response
• *What is your name?*	Open question
• *What is your institution?*	Open question
• *What is your current position?*	Open question
• *Does your institution use a structured evidence to decision/recommendation framework or process to develop public health guidelines or recommendations?*<br>○ *If 'Not sure': Since you answered that you are not sure if your institution use an evidence to decision/recommendation framework from a public health perspective, could you provide us a name and contact email of someone in your institution who might know this information?*	Yes/No/Not sure<br><br>Open question
• *Irrespective of your previous answers, do you know any additional evidence to decision/recommendation framework or process to develop public health guidelines or recommendations?*<br>○ *If your answer was "Yes", could you provide us the name, reference or example of use of that framework?*	Yes/No

• *Considering the above definitions, which frameworks are used in your institution?*<br>○ *If 'Other': Since you answered "Other" in the previous question: Which framework does your institution use for moving from evidence to a recommendation or decision from a public health perspective? (Please provide the name and/or reference) If your institution uses a self-developed or in-house framework, please state so.*	GRADE-EtD<br>WHO-INTEGRATE<br>EURECCA<br>PREVIDE<br>WICID<br>Other
• *Has your institution adapted one (or more) of these frameworks?*	Yes/No
• *Are you aware of any representative example of the use of one of these frameworks to the infectious disease field, conducted at your institution? Examples may include guidelines, policy briefs, health technology assessments, internal documents, or other documents.*<br>○ *If "Yes" in the previous question, could you provide us the name of the document and link or reference?*	Yes/No
• *How would you describe the experience of using the framework?*	Open question
• *In your opinion, what were the main barriers for its use?*	Open question
• *In your opinion, what were the main facilitators or enablers for its use?*	Open question
• *We may need to contact you for further information. Would you be willing to participate in a brief interview? If "Yes", we will contact you via email. Data derived from this interview may be used for project and publication purposes only.*<br>○ *If you selected "Yes" in the previous question, please kindly provide us with your email address.*	Yes/No<br><br>Open question

Supplement S4. Excluded studies by full-text screening, with reasons

Study ID	DOI	Reason for exclusion
Abbasian 2020	10.2147/RMHP.S258661	Not an EtD framework
Abbey 2017	10.1186/s12889-016-3957-1	Not an EtD framework
Aiassa 2022	10.14573/altex.2004211	Not an EtD framework
Akiyama 2021	10.1016/S2468-1253(20)30365-4	Not an EtD framework
Alonso-Coello 2018a	10.1016/j.gaceta.2017.03.008	Language other than English
Alonso-Coello 2018b	10.1016/j.gaceta.2017.02.010	Language other than English
Alsalem 2022	10.1007/s10462-021-10124-x	Not an EtD framework
AlSiyabi 2021a	10.1123/jpah.2021-0235	Not an EtD framework
AlSiyabi 2021b	10.1123/jpah.2021-0152	Not an EtD framework
Alva 2018	10.3390/ijerph15030522	Not an EtD framework
Ananthapavan 2021	10.1186/s12961-021-00796-w	Non-structured process
Angelis 2017	10.1016/j.socscimed.2017.06.024	Non-public health decision
Angelis 2020	10.1016/j.socscimed.2019.112595	Not an EtD framework
Association of Women's Health, Obstetric and Neonatal Nurses 2022a	10.1016/j.jogn.2022.01.001	Non-public health decision
Association of Women's Health, Obstetric and Neonatal Nurses 2022b	10.1016/j.nwh.2022.01.001	Non-public health decision
Baltussen 2017	10.1016/j.jval.2016.11.019	Does not describe domains, factors or criteria considered

Baltussen 2021	10.34172/ijhpm.2021.158	Does not describe domains, factors or criteria considered
Bao 2021	10.1186/s12913-021-06827-0	Non-public health decision
Behzadifar 2021	10.15167/2421-4248/jpmh2021.62.2.2041	Not an EtD framework
Benmarhnia 2017	10.15171/ijhpm.2017.28	Not an EtD framework
Bertone 2013	10.1186/1478-4505-11-39	Not an EtD framework
Blythe 2022	10.5334/ijic.5997	Not an EtD framework
Bowen 2016	10.2105/AJPH.2015.302970	Not an EtD framework
Brady 2016	10.1016/j.evalprogplan.2016.01.003	Not an EtD framework
Bragge 2017	10.1186/s12874-017-0314-8	Not an EtD framework
Brands 2018	10.3390/ijerph15050942	Not an EtD framework
Brindis 2014	10.1146/annurev-publhealth-032013-182455	Not an EtD framework
Brunton 2016	No DOI. https://eppi.ioe.ac.uk/CMS/Portals/0/PDF%20reviews%20and%20summaries/Employer-led%20workplace%20health%202016%20Brunton.pdf	Not an EtD framework
Backman 2022	10.34068/joe.60.02.21	Not an EtD framework
Caiaffa 2014	10.1007/s11524-013-9812-0	Not an EtD framework
Calonge 2022	10.1002/jrsm.1582	Not an EtD framework
Camps 2020	10.1200/JOP.19.00487	Not an EtD framework
Cao 2022	10.1111/jonm.13458	Not an EtD framework

Chambers 2015	10.1093/pubmed/fdu069	Not an EtD framework
Chan 2020	10.1136/bmjopen-2019-032884	Not an EtD framework
Ciro Correa 2020	10.1186/s12961-020-00588-8	Not an EtD framework
Cole 2015	10.5888/pcd12.150300	Not an EtD framework
Coles 2016	10.1111/1468-0009.12195	Not an EtD framework
Conrad 2019	10.1016/j.zefq.2019.02.006	Language other than English
Crépault 2016	10.1016/j.drugpo.2016.04.013	Not an EtD framework
Dahm 2017	10.1016/j.jclinepi.2017.02.019	Non-public health decision
Davies 2014	10.1016/j.puhe.2013.11.011	Not an EtD framework
De Pietro 2015	No DOI. PMID: 26766626	Not an EtD framework
deFolter 2018	10.1017/S0266462318000090	Non-public health decision
Dinda 2020	10.4103/ijmr.IJMR_3640_20	Not an EtD framework
Djulbegovic 2014	10.1200/JOP.2013.001364	Not an EtD framework
Dörr 2022	10.1371/journal.pone.0263898	Not an EtD framework
Escoffery 2018	10.1186/s13012-018-0815-9	Not an EtD framework
Field 2016	10.1186/s12961-016-0154-8	Not an EtD framework
Fischer 2021	10.1007/s11606-020-06451-4	Does not describe domains, factors or criteria considered
Fourn 2020	10.3917/spub.202.0273	Language other than English
FrutosPérez-Surio 2019	10.1186/s40545-019-0181-2	Not an EtD framework
Funk 2022	10.1016/j.healthpol.2021.10.001	Not an EtD framework
Gaffey 2021	10.1016/S0140-6736(21)00133-1	Not an EtD framework
Garcia 2018	10.5123/S1679-49742018000200020	Not an EtD framework

Gębska-Kuczerowska 2020	10.3390/ijerph17207657	Not an EtD framework
Glover 2020	10.1016/j.jclinepi.2020.06.004	Not an EtD framework
González-Lorenzo 2015	10.1016/j.vaccine.2014.12.020	Non-public health decision
González-Lorenzo 2016	10.1701/2152.23272	Language other than English
Grant 2022	10.1097/01.NUMA.0000874432.64403.fb	Not an EtD framework
Grill 2017	10.1007/s10728-015-0299-6	Not an EtD framework
Guo 2021	10.1186/s12875-021-01556-z	Not an EtD framework
Harder 2015	10.1016/j.healthpol.2015.02.010	Not an EtD framework
Harder 2017	10.2807/1560-7917.ES.2017.22.40.16-00620	Not an EtD framework
Hart 2022	10.2105/AJPH.2022.306929	Not an EtD framework
Hester 2022	10.1016/j.jpeds.2022.06.002	Not an EtD framework
Holly 2022	10.1002/lrh2.10295	Not an EtD framework
Inotai 2018	10.1080/14737167.2018.1508345	Not an EtD framework
IOM 2015	10.17226/19013	Does not describe domains, factors or criteria considered
Janati 2018	10.4314/ejhs.v28i3.8	Not an EtD framework
Jessani 2021	10.1186/s12961-021-00733-x	Not an EtD framework
JimenezdelaJara 2015	10.1186/0717-6287-48-10	Not an EtD framework
Jones 2017	10.1016/j.socscimed.2017.01.048	Not an EtD framework
Jones-Bonofiglio 2020	No DOI. PMID: 32880333	Not an EtD framework
Kallenbach 2019	10.1016/j.zefq.2019.06.001	Language other than English

KamphuisCBM 2022	10.1093/eurpub/ckac068	Not an EtD framework
Keygnaert 2016	No DOI. PMID: 27786434	Not an EtD framework
Kim 2019	10.1016/j.ypmed.2019.105781	Does not describe domains, factors or criteria considered
Kolasa 2018	10.1080/14737167.2018.1467759	Non-public health decision
Kuchenmüller 2022	10.1016/j.evalprogplan.2022.102053	Not an EtD framework
Kumar 2020	10.1093/heapol/czaa027	Not an EtD framework
Lalani 2018	10.1111/hex.12852	Not an EtD framework
Lane 2021	10.1108/LHS-03-2021-0013	Not an EtD framework
Lewin 2019	10.1186/s12961-019-0468-4	Not an EtD framework
Li 2017	10.12688/f1000research.10966.1	Not an EtD framework
Li 2022	10.1016/j.imr.2022.100841	Does not describe domains, factors or criteria considered
Lietz 2020	10.1016/j.zefq.2020.03.002	Language other than English
Lin 2020	10.1007/s11606-020-05783-5	Not an EtD framework
Lo 2019	10.1136/bmjopen-2018-026482	Not an EtD framework
Lotfi 2022	10.1016/j.jclinepi.2021.09.028	Not an EtD framework
Luoto 2013	10.1371/journal.pmed.1001469	Not an EtD framework
Mahdavi 2021	10.34172/ijhpm.2021.142	Not an EtD framework
Malin 2022	10.1007/s15010-021-01645-2	Non-public health decision
Maree 2021	10.1071/AH19290	Not an EtD framework
Martins 2021	10.1007/s11524-021-00560-z	Not an EtD framework
McLaren 2016	10.1002/14651858.CD010166.pub2	Not an EtD framework

McPhaul 2013	10.3912/OJIN.Vol18No01Man04	Not an EtD framework
Morche 2018	10.1016/j.zefq.2018.03.004	Language other than English
Morgan 2018	10.1016/j.jclinepi.2017.09.023	Non-public health decision
Morgano 2017	10.1701/2802.28354	Language other than English
Morgano 2018	10.1701/2902.29246	Language other than English
Mostafavi 2016	10.5539/gjhs.v8n10p212	Not an EtD framework
Murad 2020	10.1016/j.mayocp.2020.05.009	Not an EtD framework
Murphy 2021	10.3310/hta25760	Non-public health decision
Mwendera 2017	10.1186/s12961-017-0264-y	Not an EtD framework
Neale 2019	10.1093/advances/nmy113	Not an EtD framework
Neumann 2018	10.1016/j.jval.2017.12.012	Not an EtD framework
Nicod 2017	10.1007/s10198-016-0823-0	Non-public health decision
Norton 2019	10.1186/s12961-019-0463-9	Not an EtD framework
Nussbaumer-Streit 2018	10.1016/j.zefq.2018.05.004	Language other than English
Oxman 2010	10.1016/S0140-6736(09)61251-4	Published before 2013
Palazzo 2016	10.1701/2218.23926	Language other than English
Parmelli 2017	10.1017/S0266462317000447	Non-public health decision
Paulden 2015	10.1007/s40273-014-0235-x	Non-public health decision
Perfetto 2018	10.1016/j.jval.2017.12.002	Not an EtD framework
Pertschuk 2013	10.1097/PHH.0b013e3182582a57	Not an EtD framework
Pfeifflé 2019	10.3390/nu11020362	Not an EtD framework
Poulin 2013	10.2147/MDER.S51384	Not an EtD framework

Prasinos 2022	10.1109/JBHI.2022.3142503	Not an EtD framework
Qin 2020	10.1371/journal.pone.0237342	Non-public health decision
Quilodrán 2021	10.5867/medwave.2021.04.8182	Language other than English
Redman 2015	10.1016/j.socscimed.2015.05.009	Not an EtD framework
Restar 2019	10.1371/journal.pone.0224133	Not an EtD framework
Rodes-Sanchez 2022	10.1016/j.vaccine.2022.05.054	Not an EtD framework
Rütten 2016	10.1055/s-0035-1548883	Language other than English
Rycroft-Malone 2013	10.1186/1748-5908-8-28	Not an EtD framework
Sacks 2020	10.1007/s13679-020-00376-z	Not an EtD framework
Schloemer 2018	10.1186/s13012-018-0751-8	Not an EtD framework
Schoelles 2017	10.23970/AHRQEPCWHITEPAPER3	Not an EtD framework
Sculpher 2018	10.1016/j.jval.2017.12.003	Not an EtD framework
Shaban-Nejad 2017	10.3233/978-1-61499-830-3-1335	Non-structured process
Shah-Manek 2017	10.18553/jmcp.2017.23.6-a.s13	Non-public health decision
Shekelle 2013	No DOI. PMID: 23427349	Not an EtD framework
Silva 2016	10.1016/j.healthpol.2016.01.005	Not an EtD framework
Sin 2015	10.12809/hkmj144326	Not an EtD framework
Siu 2015	10.12809/hkmj144307	Not an EtD framework
Sofi-Mahmudi 2022	10.1093/heapro/daab049	Not an EtD framework
Solow 2018	10.1016/j.jval.2017.12.004	Not an EtD framework
Sosa 2021	10.1007/s10995-020-03018-x	Does not describe domains, factors or criteria considered
South 2019	10.1093/heapro/dax083	Not an EtD framework

Stafinski 2011	10.2165/11539840-000000000-00000	Non-public health decision
Tan 2019	10.1177/1355819619842305	Not an EtD framework
Thompson 2022	10.1186/s12961-022-00902-6	Not an EtD framework
Timotijevic 2013	10.1080/10408398.2012.747485	Does not describe domains, factors or criteria considered
Turner 2017	10.1001/jamapediatrics.2017.1360	Not an EtD framework
Unsworth 2021	10.1177/20552076211018617	Not an EtD framework
Vélez 2020	10.1186/s12961-020-00584-y	Not an EtD framework
Venkatesan 2019	10.1371/journal.pone.0223946	Not an EtD framework
Votruba 2021	10.1186/s12961-020-00651-4	Not an EtD framework
WaltersLEM 2018	10.2196/jmir.9940	Not an EtD framework
Weber 2017	10.1186/s12904-017-0252-6	Not an EtD framework
Wende 2022	10.1186/s43058-022-00316-z	Not an EtD framework
WHO 2013	No DOI. https://apps.who.int/iris/handle/10665/131300	Not an EtD framework
WHO 2018	No DOI. https://www.who.int/publications/i/item/9789241514088	Not an EtD framework
Wickremasinghe 2016	10.1093/heapol/czv079	Not an EtD framework
WongCHL 2020	10.1177/1534735420940418	Non-public health decision
Yazdi-Feyzabadi 2021	10.1186/s13690-021-00737-7	Not an EtD framework
Yearwood 2018	10.26633/RPSP.2018.91	Not an EtD framework
Yoder-Wise 2020	10.1111/nuf.12381	Not an EtD framework

Yue 2022	10.1186/s12913-022-07493-6	Not an EtD framework
Zawadzki 2021	10.1016/j.jval.2021.03.005	Not an EtD framework
Zucca 2021	10.3389/fpubh.2021.653588	Not an EtD framework

Supplement S5. List of included references and documents for each framework and research question

Framework	RQ1 - RQ2	RQ3	RQ4
**GRADE-EtD**	Main reference: [1]<br><br>Complementary references: [2–8]<br><br>Adaptations of the framework: [9–14]	[15–18]	[14,19–26]
**WHO-INTEGRATE**	Main reference: [27]<br><br>Complementary references: [28]	[29,30]	[31–33]
**PSE framework**	[34]	-	-
**Framework for planning and improving evidence-based practices**	[35]	-	-
**EEFA framework**	Main reference: [36]<br><br>Complementary references: [37,38]	[39,40]	-
**Framework for prioritising policy choices**	[41]	-	-
**WICID**	[42]	-	-
**EURRECA**	Main reference: [43]<br><br>Complementary references: [44]	-	-
**Ontario Decision Framework**	[45]	-	-
**Policy Framework for Technology Assessment**	[46]	-	-
**Policy Framework for Primary Prevention of Occupational Cancer**	[47]	-	-
**EVITA**	Main reference: [48]<br><br>Complementary references: [49]	-	-
**Framework of evidence-based decision-making in health system management**	[50]	-	-
**PREVIDE**	[51]	-	-

**CPSTF framework**	[52]	[53,54]	-

CPSTF: Community Preventive Services Task Force; EEFA: Ethics, Equity, Feasibility, and Acceptability; EtD: Evidence to Decision; EURRECA: EURopean micronutrient RECommendations Aligned; EVITA: EVIdence To Agenda; GRADE: Grading of Recommendations, Assessment, Development, and Evaluation; INTEGRATE: INTEGRATe Evidence; PREVIDE: PREVention decIDE; PSE: Policy, Systems, and Environmental; WHO: World Health Organization; WICID: WHO-INTEGRATE COVID-19.

Supplement S6. Detailed description of barriers and facilitators as reported by the identified literature

Reference	Topic	Overall experience	Barriers	Facilitators
GRADE EtD framework				
Guldbrandsson 2016	Public health field in Sweden.	• Positive attitudes towards the overall process<br>• Panel discussion allows perspectives' adjustment and consensus<br>• This framework helps reach consensus among panel members.<br>• There may be some language-dependent translation suggestions between English and Swedish.<br>• Two aspects were not being considered by the framework: "Individual autonomy" and "method sustainability"	**1. Complicated evidence grading system**<br>*"Evidence grading perceived as complicated."*<br>**2. Lacking evidence or inappropriateness of RCT for public health**<br>*"RCT-based decisions may not be always appropriate for public health."*<br>**3. Lack of implementation**<br>**/adaptation consideration**<br>"This framework may not address further implementation/adaptation at local or regional level."	**1. Stakeholder engagement**<br>*"Panel composition is critical, and should reflect all interests related to the recommendations or decisions."*<br>**2. Guidance**<br>*"User needs clear instructions regarding the form and the panel procedure."*<br>**3. Tailoring of factors according to context**<br>*"The framework is useful but requires tailoring to public health field."*
Friesen 2022	National food fortification programming in Nigeria	• GRADE-EtD increases the systematic use of evidence<br>• GRADE-EtD raise awareness of local evidence gaps<br>• GRADE-EtD does not directly address other factors (such as politics or social)	-	-

Li 2018	We aimed to determine whether GRADE (normative criteria) dominate non-GRADE (descriptive factors).	·GRADE EtD criteria contributes to most of the discussions within the panels<br>·GRADE EtD dominates the decision process, which may lead the panel to ignore other relevant factors.<br>·GRADE-EtD may not always explicitly include all the relevant criteria<br>·The users are inclined to be transparent about the description of the decision-making process.<br>· Clinical experience should be accompanied by research evidence<br>· The political environment can be an additional criterion for the EtD process to draw on relevant political advances and to facilitate decision-making.	**1. Lack of evidence/low-quality evidence** was a recurrent issue.	**1. Sufficient evidence** facilitates the rapid decision-making process.<br>**2. Facilitators for different factors:**<br>· *Affordability considerations at different levels and cost-effectiveness analyses as key topics for assessing resources and costs;*<br>· *Providing different scenarios and discussed on desirable and undesirable effetcs facilitate the balance of benifits and harms;*<br>· *Acceptability and feasibility may related to implementation of recommendation;*<br>· *Patient values and preference was addressed by patient representatives, supplementing with clinical expertise and experience on patient value;*<br>· *Equity was considered by addressing access to recommended interventions/tests or health coverage.*<br>**3. Previous training** facilitates the application of GRADE EtD.<br>4. **Clinical experience is useful** for brainstorming about making the best recommendations

Stalteri Mastrangelo 2021	Tuberculosis, gonorrhoea and respiratory tract infection guidelines, considering antimicrobial resistance	·GRADE enhances consideration of contextual factors	-	-
Meneses-Echavez2021	To describe users' experiences with the interactive Evidence to Decision (iEtD) framework and identify main barriers and facilitators related	·Users refer no problems making background and PICO questions<br>·Users would prefer to assess desirable and undesirable effects in one unique section instead of two separate ones<br>·Positive experiences using the Conclusions section<br>·Users value the option of tailoring the framework (e.g. limiting the number of criteria for rapid health technology assessments, or modifying the order of the criteria)	1. Additional workload required regarding evidence synthesize and preparation of presentation format.<br>2. Difficulty to coordinate framework completion among large group.<br>3. Lack of knowledge of using EtD and GRADE approach, including different factors like equity<br>4. The term "values" is perceived as confusing for some users	1. Guidance is needed

Moleman 2022	To examine guideline quality in relation to the availability of certain types of evidence and to reflect on the implications of CPGs' promise to improve the quality of care practices.	·GRADE perceived as a methodological improvement compared to other approaches, more transpartent and systematic.<br>·Some participants perceive an overemphasis in best external evidence ("scientisation")<br>· Recommendations with weak supporting evidence may be underrepresented in guideline development<br>· There may be difficulties for addressing multiple comparisons.<br>· PICO section should be more explicit<br>· "Research priorities" section seen as important	1. Difficulties applying GRADE for questions not responded by RCTs<br>2. Difficulties integrating science and other consideration<br>3. Suboptimal wording for recommendations comparing two active interventions<br>4. Framework may be too long, with sections that may not be relevant in specific circumstances<br>5. There may be overlap between some criteria<br>**6. Barriers for each factor**<br>· "Variability"" and "uncertainty" should be differentiated in "Values and preferences", and source of information should be explicit<br>· Major dificulties in the "balance of benefits and harms", with problems answeing consistently the questions about the size of the effect, and some questions considered redundant<br>· Mixed views about "Resource use", depending if the panel included health economists ("too superficial") or not (struggled answering questions)<br>· More guidance needed for answering "equity" considerations, with suggestions to add the option of "no effect on health equity"<br>· More guidance needed for "acceptability", problems for identifying relevant stakeholders<br>· More guidance needed for justification/remarks	1. GRADE separates the evidence summary and the formulation of recommendations processes, allowing to distinguish opinion-based and scientific-based recommendations.<br>2. Use of EtD framework may need previous training in GRADE methodology

			· More guidance needed for implementation considerations<br>7. Some issues regarding wording of panel decisions (recommendations)	

Rosenbaum 2018	To help decision makers achieve fairness in their decision making by creating tools that would facilitate these three process elements.	·EtD useful for structuring information and discussion<br>·EtD provides structure and facilitates management of the panel.	1. Chairs challenge: Time, domineering participants, avoiding bias in discussion and when introducing information.<br>2. Amount of information could be overwhelming.<br>3. Challenges for condense evidence presentation - skills from Chair is needed.<br>4. Inconsistency of wording can be challenging.<br>5. Some users may need explanations specific for some elements (such as "Values")<br>6. Skills required for retrieving evidence and additional considerations<br>7. Overlapping among criteria	1. A good chair is a key for a successful use of the framework.<br>2. Different levels of skills understanding evidence and numerical data.<br>3. Users prefer judgements separated, organisated, and on the same page as the summaries of evidence.<br>4. "Additional considerations" is useful for including other sources of information<br>5. Flexibility for decision making is required, which can be either easier or difficult.
Stadelmaier 2022	Nutrition-related policy-making for a European country	·EtD as structured, facilitating discussions and decision-making	-	1. Policy-makers may find it easier to understand summarised evidence in EtD framework rather than a set of SRs
**WHO-INTEGRATE**				

Murano 2022	To describe the methods used to apply WHO-INTEGRATE and present summary results of the evidence review for each of the EtD criteria for the three induction of labour topics.	·WHO-INTEGRATE allowed to explore health rights and inequity in a detailed, systematic and transparent way.		**1. Systematic mapping methods:**<br>*"Use of EtD may be enhanced by systematic mapping methods, consideration of other frameworks, and complementary work with social science researchers."*<br><br>Facilitators for each EtD factor:<br>**2. Trial-based studies for Resources used:**<br>*"selection of economic evidence to trial-based studies to avoid challenges with assessing model validity and generalizability"*<br><br>**3. For equity**<br>**3.1 Consider population characteristics and settings at early stage:** "*Inclusion of evidence from diverse populations and settings in early stages can inform considerations around equity."*<br><br>*"Providing to panel regarding population characteristics would further enhance discussion of equity, provide research agenda, and focus on future updating."*<br><br>**3.2 Including Social science equity researcher**<br>"Inclusion of social science equity researchers would be beneficial"<br><br>**4. For Decision making process:**<br>**4.1 Team collaboration**

				"Team collaboration was relevant for consistency and interpretation of findings."<br><br>**4.2. Providing a multi-layered evidence presentation format**<br><br>*"Providing high-level summary of evidence for each criterion; providing supplemental file with detailed findings of evidence; and evidence gap map for each criterion"*

Stratil 2022	To assess WHO-INTEGRATE framework comprehensiveness and usefulness for public health and health policy decision-making	·WHO-INTEGRATE seen as useful and comprehensive<br>·All WHO-INTEGRATE criteria was seen as important, and none should be dropped.<br>·WHO-INTEGRATE framework separates individual and population perspectives, range of feasibility considerations, broad perspective beyond health implications<br>·Participants think that several criteria and subcriteria need modifications on wording and definition, and have missing aspects, while some few others need order and grouping, or have overlap, redundancy or need delineation<br>·Users fell the framework succesfully covered their reasoning.<br>·Using WHO-INTEGRATE can be overwhelming due to complexity and additional workload. This can lead to skipping important domains.	1. **More guidance is needed:**<br>*"Users feel more guidance is needed for using WHO-INTEGRATE."*<br>2. **Skipping domains** to reduce workload may diminish the value of the final product. Appropriate resources to conduct a guideline are necessary.<br>3. Limited by **following the same approach as the GRADE EtD framework** (defining as intervention - gather evidence - make recommendation).<br>4. WHO-Integrate should focus on beneficiaries and asking what should be done to improve health and well-being.<br>**5. Limited availability and low certainty of evidence:**<br>Identifying evidence for some criteria might be challenging.<br>**6. Context-dependence may limit applicability:**<br>"Many aspects of WHO-INTEGRATE are context-dependent, which limits its applicability for global guidelines."<br>7. "Societal implications" was perceived as fuzzy and vague<br>8. **Barriers for EtD factors:** blurry boundaries between several criteria and sub-criteria.	NR

			8.1. Value as a sub-criterion for "Patients'/beneficiaries" may not receive enough attention<br><br>8.2. Human rights and acceptability considerations should be separated into two distinct criteria.<br><br>8.3. Non-discrimination could be under Human rights instead of Equity and equality<br><br>8.4. Combine societal impact and health impact into one broad impact-oriented criterion<br><br>**9. Possible missing aspects:** Intervention sustainability, reliability and quality of an intervention, outcomes related to well-being, political feasibility	

Webnitz 2023	To identify lessons learnt for similar endeavours by addressing the following research question: What were the strengths and weaknesses of the guideline development process as perceived by the different groups involved?	·EtD provided a comprehensible structure and transparency for making recommendations, especially in abscence of conclusive evidence<br><br>·EtD helped the panel to ground recommendations in reality and to consider potential side effects<br><br>·WHO-INTEGRATE criteria was mainly applied in working group of scientists, not so much in full group meetings with practitioners and school family members.<br><br>·WHO-INTEGRATE framework allowed health and societal implications to be considered systematically, mostly informed by anecdotal expertise due to lack of studies and of professional experise.<br><br>·Consequences beyond health and education were not systematically considered<br><br>·Some questioned the added value of using the WHO-INTEGRATE EtD, mainly referring to their suitablity for practical considerations about panel members' voting behaviour, guideline acceptability, and implementation	**1. Methods-related decision process**:<br><br>"Critics about methods-related decision process, such as the decision of choosing a preferred option after initial voting (instead of voting again), or not formally prioritizing endpoints for outcomes."<br><br>Barrier:<br><br>**2. High number of topic areas** for recommendations may deprive the panel of time and resources to discuss fewer recommendations more in depth.<br><br>**3. Unclear how panel member should be selected**<br><br>**4. The choice of evidence may cause criticism on decision making process**, for example using modelling studies but refusing lab-based studies.<br><br>5. Barriers for EtD factors:<br><br>**5.1 Value and preference:** Lack of qualitative research on values and preferences was a limitation<br><br>**5.2 Societal implications:** evidence and specific expertise for assessing societal implications and unintended consequences (beyond direct health impact) was missing<br><br>6. Barriers for decision making process:<br><br>**6.1 Balance different perspectives** "Main tension during recommendation development was to balance different perspectives (infectious disease control and educational perspective);"<br><br>**6.2 Balance different criteria**	1. Participants appreciate **transparent, democratic and anonymous consensus-building procedures**<br><br>2. Participants appreciate **previous identification and appraisal of scientific literature.**<br><br>3. **Sequence of process** allows work in small groups plus full-panel consensus voting, which was efficient and goal-oriented<br><br>**4. Prior assessment and tailoring of the framework** might be beneficial: *"The same participant noted that allocating more time to a thorough and comprehensive process of prioritizing and then adapting the generic criteria of the WHO-INTEGRATE framework at the beginning of the process might have been beneficial."*<br><br>**5. Evidence seen as critical in the process, and Expert opinion as crucial:** *"It is important to provide more opportunities to develop a shared understanding of evidence and its role;" "Expert opinion as crucial especially when there is a lack of evidence."*<br><br>*"In the abscence of evidence, lived experience was important (for scientists and non-scientists). Professional experience, academic credentials and eloquence may make opinions too influential."*<br><br>**6. Panel members' expertise**<br><br>*"Different types of expertise:*<br><br>*- Scientific expertise (grounded in scientific studies and disciplinary knowledge)*

			"In the working groups, some criteria may have received more attention than others (e.g. unintended health consequences and social outcomes versus economic, ecological or legal aspects);"<br><br>**6.3 Hierachy of panel members**<br><br>Some participants may dominate the discussion and have too much influence in the final results;<br><br>**6.4 Lack of experience** on guideline development might reduce possibilities for fully participation in the process<br><br>**6.5 Conflict of interest:**<br><br>"Institutional interests influence participants' arguments"	*- Practical expertise (derived from implementing school measures)*<br><br>*- Lived experience (being affected by those measures)*

Supplement S7. Detailed findings of the users' experience with the GRADE EtD framework

Overall perception of the GRADE EtD framework

Six studies exploring the experience of using the GRADE EtD framework described an overall positive perception of the process [14,19,21,22,24,25]. The GRADE-EtD framework was perceived as a structured, comprehensive and transparent approach that increased the systematic use of evidence, and facilitated discussion and decision-making [14,19,21–25]. One study observed that policy-makers may find it easier to understand summarised evidence using a GRADE EtD framework, rather than using a set of systematic reviews [24]. In another study, users reported mixed views about the level of detail of the framework, with some preferring simpler solutions, while others advocating for more complexity [19]. Besides evidence on health effects, the GRADE EtD framework enhances the consideration of other factors, such as acceptability, feasibility or contextual factors [19,26].

Panel composition and workflow

Users perceived the panel composition of important stakeholders as critical, highlighting the importance of reflecting all interests related to the recommendations or the final decisions [14]. Panels may have members with different levels of skills related to understanding evidence and numerical data, but several users emphasised that having a trained and knowledgeable chair for the panel was a key factor for a successful use of the framework [19]. The main reported challenges for the chair were time management, dealing with domineering participants, and avoiding bias when introducing information or discussing [19].

Panel discussion allowed perspectives' adjustment and facilitated consensus [14]. Nevertheless, the workflow among panels using the GRADE EtD is variable [19]. Clinical experience was perceived as useful for brainstorming into the discussion about making the

best recommendation, although users reported that it should always be accompanied by research evidence, if available [20].

Assessing the evidence in the public health field

Users referred that randomised controlled trials are scarce and may not always be the appropriate research design in the public health field, which can make the appraisal of the evidence as proposed by the GRADE methodology more difficult [14,25]. Other studies (not specifically assessing the experience of using the framework itself) have also highlighted similar problems when assessing the certainty of evidence for public health using the GRADE methodology [55,56].

Experience using GRADE EtD framework's criteria

Most of the panels' discussions were related to reviewing the research evidence to determine the effects of an intervention [20]. If evidence was sufficient and clear, the decision-making process was rapid, and it took longer if no evidence or only low-quality evidence was available [20].

The study by Neumann et al showed that "values and preferences" and "balance of benefits and harms" criteria, posed difficulties for several panels, mainly due to the differentiation between "uncertainty" and "variability", and the difficulty to judge the magnitude of desirable and undesirable effects, and its relationship [21]. Panels struggled to answer the questions related to "balance of benefits and harms" consistently, with a few methodologists identifying some questions as redundant [21]. The current GRADE EtD version has already been updated to address most of these challenges [21].

Some participants found terms such as "values" and "equity" to be confusing, expliciting a need for more guidance to assess them [19,21,22]. In the "equity" criterion, some suggested adding the option "no effect on health equity" as an answer [21]. There were mixed views about the "resource use" criterion: Panels without health economists struggled to assess it, while panels with health economists considered the criterion too superficial [21].

A possible overlap between some criteria was mentioned (specifically between "acceptability" and "feasibility" with "values and preferences" and "resource consideration"), and users may have poor understanding of specific criteria and the overall GRADE approach [21,22]. Some teams expressed not being sure what criterion an issue belonged to [19]. Some users also perceived a process of "scientisation", related to the overemphasis made in best external evidence, and difficulties in integrating it with the experience. However, separating the evidence summary and the formulation of recommendations was positively perceived, as it allowed to distinguish opinion-based from scientific-based recommendations [25].

The value of tailoring the framework

Users value the option of tailoring the framework in different ways [14,19,22,23]. Users may feel the framework to be too long for specific circumstances, containing sections that may not be always relevant [21]. Users value the option of tailoring the framework by limiting the number of criteria, modifying the order of the criteria, or changing the judgement options [19,22].

Authors have also described that the GRADE EtD framework's criteria may not directly address all the relevant factors for specific decision-making processes, which may lead to a need of including new considerations [14,22,23]. For example, Friesen et al reported that the GRADE EtD framework does not directly assess political or social factors [23]; Guldbrandsson et al decided to tailor the framework, incorporating two new questions about "individual autonomy" and "method sustainability" for the Swedish public health context [14]; and Li et al identified legal context as a non-explicit GRADE criterion [20].

The need for training

The use of the GRADE EtD framework needs previous training in overall GRADE methodology or in the use of specific tools (e.g. iEtD) [21,22]. Some challenges may arise at the moment of searching or presenting evidence, or about what to do when no evidence is available [19]. As stated before, the assessment of specific criteria may also need

guidance or previous training, such as "equity", "acceptability" or "values and preferences" [21].

When using the GRADE framework, the vast majority of the panels' discussions were related to the frameworks' predefined criteria [20]. This could be due to the criteria really being comprehensive enough, or also due to the influence of previous training in the GRADE approach itself [20].

Importance of language and wording

Wording and language were important issues in several aspects. Some users stated that the wording of terminology and signalling questions from the GRADE EtD framework in the assessment section was unclear [22]. In order to be transparent and enhance communication, the wording of recommendations represented an important part of the panels' discussions [19,20], and, in some cases, users reported suboptimal wording for recommendations comparing two active interventions instead of one active intervention versus placebo or no treatment [21]. In some contexts, the framework may need to be tailored specifically due to language-dependent issues [14].

Experience with iEtD tool

The overall experience using an iEtD was positive, with users describing the tool as intuitive, simple, easy-to-use, well-organised, and freely available [19,22]. Users appreciated the help sections and the distinction between evidence and judgements/additional considerations [19,22]. Users reported mostly positive experiences for formulating the PICO question and background; for assessing the criteria; and for making the conclusions [22]. The interactive online voting option was also highly valued [19,22].

Drawbacks described by iEtD users were related to the additional workload when working in large groups or with large amounts of evidence; preference for finishing the work offline; using more familiar software (such as Microsoft Word or Excel); and specific aspects of the interface (e.g. problems inserting 'summary of findings' tables, preference for assessing

desirable and undesirable effects in one section instead of two, or preference for horizontal format rather than a vertical one) [22]. Some people manifested concerns about the security and ownership of the work completed, being an online tool [19]. Due to the burden of having to learn a new, unfamiliar technology, some people still preferred to use paper [19].

Supplement S8. Detailed findings of the users' experience with the WHO-INTEGRATE EtD framework

Overall perception of the WHO-INTEGRATE framework

Three studies including 26 participants described the overall experience of using the WHO-INTEGRATE framework as positive. The framework was usually perceived as useful, detailed, structured, systematic and transparent [31–33]. Users reported that WHO-INTEGRATE allowed the separation of different perspectives (e.g. individual and population perspective), and the consideration of feasibility and broad implications beyond health [31].

However, some concerns were raised about the framework's added value [31,33]. Some users pointed out that the framework was too comprehensive, which may affect its use [31]. Some concerns were raised about WHO-INTEGRATE framework's practical considerations, in terms of panel members' voting behaviour, guideline acceptability, and implementation [33]. For example, some participants perceived the framework as too complex and questioned whether the working groups assessment of each criteria was really relevant for the voting members [33]. Users perceived that more guidance is still needed [31].

Panel members' profiles, roles and hierarchy

The inclusion of different profiles in the developing team was positively perceived, with team collaboration providing consistency and better interpretation of findings, and diversity of perspective providing legitimacy [32,33]. Some users recommended the inclusion of legal experts within the team [31], and researchers considered that the inclusion of social science researchers may be valuable for reinforcing theoretical understanding, methods, and interpretation of the evidence [32].

One study describing the experience of guideline development for school measures during the COVID-19 pandemic deepened on the understanding of the roles, profiles, and

hierarchies within the developing teams [33]. In this study and due to time pressure, secretariat members were involved in most of the development process (beyond the roles of coordination and methods support), assigning different roles for different participants, which evoked diverging opinions [33].

Three types of expertise were described in this guideline development, which ultimately defined each member profile: i) scientific expertise, grounded in scientific studies and disciplinary knowledge; ii) practical expertise, derived from implementing the school measures; and iii) lived experience of those affected by the measures [33]. The profiles influenced the differential insight about how to consider or interpret different types of evidence and outcomes [33]. Since the guideline secretariat decided who to invite and what functions to assign, this role was decisive [33]. Some members perceived a lack of transparency in terms of panel composition (i.e. selection of institutions invited), prioritisation of endpoints, and the application of the framework itself, with lived experience experts incorporated late in the process, while recommendations were developed in working groups only with the participation of scientists [33].

Most participants reflected that there was a hierarchy present among the panel members, which was influenced by the member profile, seniority, academic credentials, professional experience, institution, and eloquence [33]. The hierarchy may give some participants too much dominance during discussions, influencing the final results, while, at the same time, institutions may influence the participants' arguments [33]. However, for some participants there were no differences between them and other panel members, feeling that their expertise was sought and appreciated [33].

The concept and consideration of evidence

Murano et al reported that, in their experience, the iterative use of WHO-INTEGRATE allowed them to become more familiar with the framework, which facilitated the extraction of the most relevant findings for decision-making [32]. Users appreciated and perceived as time-efficient the previous identification and summaries of the evidence [32,33].

Authors reflected that three outputs should be provided to the panel: i) a high level summary of evidence for each criterion; ii) a supplemental file with detailed findings of evidence; and iii) an evidence gap map for each criterion [32].

Participants agreed that evidence is critical for the guideline development process, but users with different profiles reported mixed views about how to weigh or consider different types of evidence, emphasising the need for developing a shared understanding of the concept and role of evidence [33]. Not having a common understanding about evidence led in some cases to criticism, regarding the decisions about which evidence to consider (e.g. including modelling studies but no basic research studies) [33]. Some participants highlighted that focusing on high quality quantitative evidence of effectiveness may not be feasible for complex interventions [31]. The lack of availability of directly relevant empirical studies (which may be due to ethical and other feasibility issues), the lag between study conduction and its inclusion in systematic reviews, and not considering qualitative evidence were identified among the limitations of the role of evidence in the COVID-19 pandemic context [33].

Authors propose that different search strategies may be needed for areas with limited evidence coverage (e.g. equity, feasibility, values and preferences, unintended consequences, beyond direct health impact), with an evidence map informing discussion and identifying gaps for specific populations and settings [32,33]. Being flexible in terms of identification and selection of qualitative synthesis was positively seen, as it allowed to fill previously identified evidence gaps, but, on the other hand, being restrictive in the selection of economic studies allowed avoiding challenges related to the assessment of models' validity and generalisability [32]. The consideration of qualitative evidence from diverse populations in early stages could better inform considerations about equity (e.g. planning how to face lack of evidence from low and middle income countries), providing also a research agenda and helping to focus on future recommendations updates [32]. WHO-INTEGRATE was perceived as a comprehensive, structured and transparent framework to develop recommendations even in the absence of conclusive evidence [33].

Experience using WHO-INTEGRATE criteria

Overall, all the WHO-INTEGRATE criteria and subcriteria were seen as important, relevant and comprehensive for real-world public health decision-making, and users thought none should be dropped [31]. However, the complexity and additional workload, of actually using the framework, may lead to skipping (or prioritising) some domains, which may diminish the value of the final product [31,33].

Some issues with wording and definitions were reported, specifically in the "equity, equality and non-discrimination" and "societal implications" criteria [31]. For example, the criterion "societal implications" was perceived as fuzzy and vague [31]. Some users perceived missing aspects in the "balance of benefits and harms", "human rights and socio-cultural acceptability", and "equity, equality and non-discrimination" criteria [31]. Possible missing aspects reported were related to the sustainability of the intervention, the reliability and quality of the intervention, the consideration of wellbeing-related outcomes, and political feasibility [31]. Also, some participants stated that the framework might not be sufficient for reflecting underserved populations or vulnerable groups [31]. However, several users refer that no relevant criterion is missing in the framework, and the WHO-INTEGRATE developers stated that criteria identified as missing were actually covered by the framework, although there was room for improvement in terms of wording and clarification [31].

Other problems were reported regarding the order and grouping of the "human rights and socio-cultural acceptability" and "societal implications" criteria [31]. For example, many were concerned about "patients'/beneficiaries' values in relation to health outcomes'' being only a subcriterion, since it may not receive enough attention [31]. Other recommendations included separating human rights and acceptability into two different criteria; moving "non-discrimination" to the human rights consideration (instead of equity and equality); and combining societal impact and health impact into one broad impact-oriented criterion [31]. Participants also reported overlap, redundancy or delineation problems for several criteria and subcriteria [31]. For example, the boundaries between

the criterion “Health equity, equality and non-discrimination” and the sub-criterion “Social impact”, or between the criterion “Financial and economic considerations” and the sub-criterion “Interaction with and impact on the health system” were perceived as blurry [31].

Focus groups that used the framework felt that it successfully encompassed their reasoning through the discussion of all criteria, despite perceiving that the assessment of some criteria was superficial, and that they did not always address each specific subcriteria [31,33]. Finally, some participants pointed out that all the new subcriteria provided by the WHO-INTEGRATE framework could also be addressed as part of the GRADE EtD framework [31].

Perspectives on the development process

Users described the identification of evidence as a challenge, especially for specific domains (e.g. health systems and feasibility considerations, financial and economic considerations, societal impact) [31]. In case of absence of evidence, lived experience was considered important [33]. Users also noted that many aspects of the WHO-INTEGRATE framework were context-dependent, which could limit the applicability of the recommendations or decisions in global guidelines [31]. The understanding of the included and excluded populations and settings is needed for discussions about equity [32]. Also, some participants questioned if involving lived experience experts from the beginning of the process could have an impact in addressing issues such as feasibility or acceptability [33]. In this sense, one participant perceived that WHO-INTEGRATE followed a similar approach than the GRADE EtD framework (defining intervention / gathering evidence / making recommendations), and proposed an approach more focused on beneficiaries by asking them what should be done to improve health and well-being [31].

Time pressures may play an important role, limiting the assessment of all concerns within the panel [33]. Users felt discontent when there was little time for sharing information and comments to prepare meetings [33]. Time pressure and lack of resources may provoke a

burden to some members due to excessive task assignments, and also may hinder the in depth discussion of the recommendations [33].

Regarding the workflow, users reported that the iterative process of working in small groups plus subsequent full-panel consensus was efficient and goal-oriented, appreciating transparent, democratic and anonymous consensus-building procedures [33]. Some specific criticisms included methods-related issues (e.g. choosing a preferred option after initial voting, instead of voting again; not formally prioritising endpoints for outcomes), the uneven consideration of each criterion, and the unequal influence of opinions among participants (related to their profile and experience in guideline development) [31,33].

Participants recognised that balancing different perspectives within the panel was challenging [33]. For example, when developing school measures for COVID-19, the guideline members had to consider the infectious disease control perspective as well as the educational perspective [33]. Although consequences beyond health and education were not systematically considered, common sense was described as important for assessing implications beyond health impacts, and agreeing on the strength of recommendations, especially in the absence of evidence [33].

Classified as ECDC NORMAL

Supplement S9. Detailed responses to the survey, with verbatims.

Participants reflected about the evidence to be considered when using the GRADE EtD framework, referring the lack of evidence as a challenge for the public health field:

- *"[The experience of using the framework is] difficult because often there is not enough scientific evidence and we are obliged to use practice based consensus in our guidelines." (Participant #1)*
- *"GRADE is very challenging to apply in Public Health settings, because in the majority of cases the certainty of evidence is low or very low, and will always be so. But action is required. I am extremely concerned that decision makers will choose not to act when certainty is low or very low, and reallocate [resources] towards more clinical treatment if there is greater certainty." (Participant #2)*
- *"(...) a key barrier was the absence of evidence (...) and the inability for this approach to handle other forms of research evidence beyond typical intervention study types." (Participant #6)*
- *"Problems with GRADE EtD for public health guidelines include (...) the fact that evidence in this space is unlikely to involve RCTs and so most studies are going to end up low/very low [certainty]" (Participant #9)*

Other participants reflected on the practical implications about the use of the GRADE EtD framework. One referred that the framework was *"Incredible useful" (Participant #13)*. Other participants expressed difficulties using it in contexts where rapid decision-making is needed, and questioned if the criteria were comprehensive and adequate to guide panel discussions in the public health field:

- *"(...) we basically found it too rigorous and time-consuming to use, as we needed rapid collection of evidence for rapid decision-making." (Participant #10)*

- *"(...) methods in GRADE are not elaborated and functional for modelling studies, data from lab and sociological studies of impact in society and more" (Participant #10)*
- *"The discussions held by the guideline panel were far more wide-ranging than could be adequately captured in the EtD." (Participant #6)*
- *"GRADE does not consider societal implications. The GRADE EtD is too complex and was developed for clinical decision-making, not public health decision-making. You can't force a round peg into a square hole." (Participant #9)*

Some participants highlighted the need for specialised knowledge for using the GRADE EtD framework:

- *"These instruments, including GRADE and EtD are fine if you have enough methodologically skilled people with a lot of time who are willing to serve the needs of rapid decision-making." (Participant #10)*
- *"There is need for a lot of training for infectious disease experts to use them (...)" (Participant #10)*
- *"[The main barrier for its use is the] learning curve" (Participant #13)*

The main facilitator for its use was the perception of the process as structured and well accepted:

- *"A framework is necessary to ensure that the factors that contributed to the final recommendation are transparently reported and justified. When guidelines are high profile the readers need to know exactly how you reached the conclusion you reached." (Participant #9)*
- *"[The main facilitator or enabler for its use is] the structured process" (Participant #13)*

References

1. Moberg J, Oxman AD, Rosenbaum S, Schünemann HJ, Guyatt G, Flottorp S, et al. The GRADE Evidence to Decision (EtD) framework for health system and public health decisions. Health Res Policy Syst [Internet]. 2018 May 29;16(1):45. Available from: http://dx.doi.org/10.1186/s12961-018-0320-2

2. Alonso-Coello P, Schünemann HJ, Moberg J, Brignardello-Petersen R, Akl EA, Davoli M, et al. GRADE Evidence to Decision (EtD) frameworks: a systematic and transparent approach to making well informed healthcare choices. 1: Introduction. BMJ [Internet]. 2016 Jun 28;353:i2016. Available from: http://dx.doi.org/10.1136/bmj.i2016

3. Alonso-Coello P, Oxman AD, Moberg J, Brignardello-Petersen R, Akl EA, Davoli M, et al. GRADE Evidence to Decision (EtD) frameworks: a systematic and transparent approach to making well informed healthcare choices. 2: Clinical practice guidelines. BMJ [Internet]. 2016 Jun 30;353:i2089. Available from: http://dx.doi.org/10.1136/bmj.i2089

4. Andrews J, Guyatt G, Oxman AD, Alderson P, Dahm P, Falck-Ytter Y, et al. GRADE guidelines: 14. Going from evidence to recommendations: the significance and presentation of recommendations. J Clin Epidemiol [Internet]. 2013 Jul;66(7):719–25. Available from: http://dx.doi.org/10.1016/j.jclinepi.2012.03.013

5. Andrews JC, Schünemann HJ, Oxman AD, Pottie K, Meerpohl JJ, Coello PA, et al. GRADE guidelines: 15. Going from evidence to recommendation-determinants of a recommendation's direction and strength. J Clin Epidemiol [Internet]. 2013 Jul;66(7):726–35. Available from: http://dx.doi.org/10.1016/j.jclinepi.2013.02.003

6. Schünemann HJ, Mustafa R, Brozek J, Santesso N, Alonso-Coello P, Guyatt G, et al. GRADE Guidelines: 16. GRADE evidence to decision frameworks for tests in clinical practice and public health. J Clin Epidemiol [Internet]. 2016 Aug;76:89–98. Available from: http://dx.doi.org/10.1016/j.jclinepi.2016.01.032

7. Schünemann HJ, Wiercioch W, Brozek J, Etxeandia-Ikobaltzeta I, Mustafa RA, Manja V, et al. GRADE Evidence to Decision (EtD) frameworks for adoption, adaptation, and de novo development of trustworthy recommendations: GRADE-ADOLOPMENT. J Clin Epidemiol [Internet]. 2017 Jan;81:101–10. Available from: http://dx.doi.org/10.1016/j.jclinepi.2016.09.009

8. Piggott T, Brozek J, Nowak A, Dietl H, Dietl B, Saz-Parkinson Z, et al. Using GRADE evidence to decision frameworks to choose from multiple interventions. J Clin Epidemiol [Internet]. 2021 Feb;130:117–24. Available from: http://dx.doi.org/10.1016/j.jclinepi.2020.10.016

9. ACIP Evidence to Recommendation User's Guide [Internet]. Advisory Committee on Immunization Practice (ACIP); 2020 Oct [cited 2023 Jun 2]. Available from: https://www.cdc.gov/vaccines/acip/recs/grade/downloads/acip-evidence-rec-frame-user-guide.pdf

10. Lee G, Carr W, ACIP Evidence-Based Recommendations Work Group. Updated Framework for Development of Evidence-Based Recommendations by the Advisory Committee on Immunization Practices. Morbidity and Mortality Weekly Report [Internet]. 2018 Nov 16 [cited 2023 Jun 2];67(45):1271–2. Available from: https://www.cdc.gov/mmwr/volumes/67/wr/pdfs/mm6745a4-H.pdf

11. Ahmed F. U.S. Advisory Committee on Immunization Practices (ACIP) Handbook for Developing Evidence-based Recommendations [Internet]. Centers for Disease Control and Prevention; 2013 Nov [cited 2023 Jun 2]. Available from: https://www.cdc.gov/vaccines/acip/recs/grade/downloads/handbook.pdf

12. Something went wrong [Internet]. [cited 2023 Jun 2]. Available from: https://idsociety.sharepoint.com/sites/CAPG/Shared%20Documents/Forms/AllItems.aspx?id=%2Fsites%2FCAPG%2FShared%20Documents%2FPractice%20Guidelines%2FInternal%20Guideline%20Policies%20and%20Procedures%2FHandbook%2FIDSA%20Handbook%20for%20CPG%20Development%202021%2D01%2D19%2Epdf&pa

rent=%2Fsites%2FCAPG%2FShared%20Documents%2FPractice%20Guidelines%2FInternal%20Guideline%20Policies%20and%20Procedures%2FHandbook&p=true&ga=1

13. Kroke A, Schmidt A, Amini AM, Kalotai N, Lehmann A, Haardt J, et al. Dietary protein intake and health-related outcomes: a methodological protocol for the evidence evaluation and the outline of an evidence to decision framework underlying the evidence-based guideline of the German Nutrition Society. Eur J Nutr [Internet]. 2022 Jun;61(4):2091–101. Available from: http://dx.doi.org/10.1007/s00394-021-02789-5

14. Guldbrandsson K, Stenström N, Winzer R. The DECIDE evidence to recommendation framework adapted to the public health field in Sweden. Health Promot Int [Internet]. 2016 Dec;31(4):749–54. Available from: http://dx.doi.org/10.1093/heapro/dav060

15. Barlam TF, Cosgrove SE, Abbo LM, MacDougall C, Schuetz AN, Septimus EJ, et al. Implementing an Antibiotic Stewardship Program: Guidelines by the Infectious Diseases Society of America and the Society for Healthcare Epidemiology of America. Clin Infect Dis [Internet]. 2016 May 15;62(10):e51–77. Available from: http://dx.doi.org/10.1093/cid/ciw118

16. Iversen BG, Vestrheim DF, Flottorp S, Denison E, Oxman AD. COVID-19-EPIDEMIC : Should individuals in the community without respiratory symptoms wear facemasks to reduce the spread of COVID-19?–a rapid review [Internet]. Norwegian Institute of Public Health; 2020. Available from: https://www.fhi.no/globalassets/dokumenterfiler/rapporter/2020/should-individuals-in-the-community-without-respiratory-symptoms-wear-facemasks-to-reduce-the-spread-of-covid-19-report-2020.pdf

17. WHO Guidelines for malaria [Internet]. World Health Organization; 2023 Mar. Available from: https://www.who.int/publications/i/item/guidelines-for-malaria

18. de With K, Allerberger F, Amann S, Apfalter P, Brodt HR, Eckmanns T, et al. Strategies to enhance rational use of antibiotics in hospital: a guideline by the German Society for Infectious Diseases. Infection [Internet]. 2016 Jun;44(3):395–439. Available from: http://dx.doi.org/10.1007/s15010-016-0885-z

19. Rosenbaum SE, Moberg J, Glenton C, Schünemann HJ, Lewin S, Akl E, et al. Developing Evidence to Decision Frameworks and an Interactive Evidence to Decision Tool for Making and Using Decisions and Recommendations in Health Care. Glob Chall [Internet]. 2018 Sep;2(9):1700081. Available from: http://dx.doi.org/10.1002/gch2.201700081

20. Li SA, Alexander PE, Reljic T, Cuker A, Nieuwlaat R, Wiercioch W, et al. Evidence to Decision framework provides a structured "roadmap" for making GRADE guidelines recommendations. J Clin Epidemiol [Internet]. 2018 Dec;104:103–12. Available from: http://dx.doi.org/10.1016/j.jclinepi.2018.09.007

21. Neumann I, Brignardello-Petersen R, Wiercioch W, Carrasco-Labra A, Cuello C, Akl E, et al. The GRADE evidence-to-decision framework: a report of its testing and application in 15 international guideline panels. Implement Sci [Internet]. 2016 Jul 15;11:93. Available from: http://dx.doi.org/10.1186/s13012-016-0462-y

22. Meneses-Echavez JF, Rosenbaum S, Rada G, Flottorp S, Moberg J, Alonso-Coello P. Users' experiences with an interactive Evidence to Decision (iEtD) framework: a qualitative analysis. BMC Med Inform Decis Mak [Internet]. 2021 May 25;21(1):169. Available from: http://dx.doi.org/10.1186/s12911-021-01532-8

23. Friesen VM, Mbuya MNN, Wieringa FT, Nelson CN, Ojo M, Neufeld LM. Decisions to Start, Strengthen, and Sustain Food Fortification Programs: An Application of the Grading of Recommendations Assessment, Development, and Evaluation (GRADE) Evidence to Decision (EtD) Framework in Nigeria. Curr Dev Nutr [Internet]. 2022 Mar;6(3):nzac010. Available from: http://dx.doi.org/10.1093/cdn/nzac010

24. Stadelmaier J, Rehfuess EA, Forberger S, Eisele-Metzger A, Nagavci B, Schünemann HJ, et al. Using GRADE Evidence to Decision frameworks to support the process of health policy-making: an example application regarding taxation of sugar-sweetened beverages. Eur J Public Health [Internet]. 2022 Nov 28;32(Suppl 4):iv92–100. Available from: http://dx.doi.org/10.1093/eurpub/ckac077

25. Moleman M, Jerak-Zuiderent S, van de Bovenkamp H, Bal R, Zuiderent-Jerak T. Evidence-basing for quality improvement; bringing clinical practice guidelines closer to their promise of improving care practices. J Eval Clin Pract [Internet]. 2022 Dec;28(6):1003–26. Available from: http://dx.doi.org/10.1111/jep.13659

26. Stalteri Mastrangelo R, Santesso N, Bognanni A, Darzi A, Karam S, Piggott T, et al. Consideration of antimicrobial resistance and contextual factors in infectious disease guidelines: a systematic survey. BMJ Open [Internet]. 2021 Jul 30;11(7):e046097. Available from: http://dx.doi.org/10.1136/bmjopen-2020-046097

27. Rehfuess EA, Stratil JM, Scheel IB, Portela A, Norris SL, Baltussen R. The WHO-INTEGRATE evidence to decision framework version 1.0: integrating WHO norms and values and a complexity perspective. BMJ Glob Health [Internet]. 2019 Jan 25;4(Suppl 1):e000844. Available from: http://dx.doi.org/10.1136/bmjgh-2018-000844

28. Stratil JM, Baltussen R, Scheel I, Nacken A, Rehfuess EA. Development of the WHO-INTEGRATE evidence-to-decision framework: an overview of systematic reviews of decision criteria for health decision-making. Cost Eff Resour Alloc [Internet]. 2020 Feb 11;18:8. Available from: http://dx.doi.org/10.1186/s12962-020-0203-6

29. Guidelines on sanitation and health [Internet]. World Health Organization; 2018. Available from: https://www.who.int/publications/i/item/9789241514705

30. 027- ARN. S3-Guideline Measures for the prevention and control of SARS-CoV-2 transmission in schools | Living Guideline. AWMF; 2022 Sep.

31. Stratil JM, Paudel D, Setty KE, Menezes de Rezende CE, Monroe AA, Osuret J, et al.

Advancing the WHO-INTEGRATE Framework as a Tool for Evidence-Informed, Deliberative Decision-Making Processes: Exploring the Views of Developers and Users of WHO Guidelines. Int J Health Policy Manag [Internet]. 2022 May 1;11(5):629–41. Available from: http://dx.doi.org/10.34172/ijhpm.2020.193

32. Murano M, Chou D, Costa ML, Turner T. Using the WHO-INTEGRATE evidence-to-decision framework to develop recommendations for induction of labour. Health Res Policy Syst [Internet]. 2022 Nov 7;20(1):125. Available from: http://dx.doi.org/10.1186/s12961-022-00901-7

33. Wabnitz K, Rueb M, Pfadenhauer LM, Strahwald B, Rehfuess EA. Rapid development of an evidence- and consensus-based guideline for controlling transmission of SARS-CoV-2 in schools during a public health emergency - A process evaluation. Front Public Health [Internet]. 2023 Mar 30;11:1075210. Available from: http://dx.doi.org/10.3389/fpubh.2023.1075210

34. Lyn R, Aytur S, Davis TA, Eyler AA, Evenson KR, Chriqui JF, et al. Policy, systems, and environmental approaches for obesity prevention: a framework to inform local and state action. J Public Health Manag Pract [Internet]. 2013 May-Jun;19(3 Suppl 1):S23–33. Available from: http://dx.doi.org/10.1097/PHH.0b013e3182841709

35. Spencer LM, Schooley MW, Anderson LA, Kochtitzky CS, DeGroff AS, Devlin HM, et al. Seeking best practices: a conceptual framework for planning and improving evidence-based practices. Prev Chronic Dis [Internet]. 2013 Dec 12;10:E207. Available from: http://dx.doi.org/10.5888/pcd10.130186

36. Ismail SJ, Hardy K, Tunis MC, Young K, Sicard N, Quach C. A framework for the systematic consideration of ethics, equity, feasibility, and acceptability in vaccine program recommendations. Vaccine [Internet]. 2020 Aug 10;38(36):5861–76. Available from: http://dx.doi.org/10.1016/j.vaccine.2020.05.051

37. Ismail SJ, Langley JM, Harris TM, Warshawsky BF, Desai S, FarhangMehr M. Canada's

National Advisory Committee on Immunization (NACI): evidence-based decision-making on vaccines and immunization. Vaccine [Internet]. 2010 Apr 19;28 Suppl 1:A58–63. Available from: http://dx.doi.org/10.1016/j.vaccine.2010.02.035

38. Ismail SJ, Tunis MC, Zhao L, Quach C. Navigating inequities: a roadmap out of the pandemic. BMJ Glob Health [Internet]. 2021 Jan;6(1). Available from: http://dx.doi.org/10.1136/bmjgh-2020-004087

39. An Advisory Committee Statement (ACS) National Advisory Committee on Immunization (NACI): Updated Recommendations on the Use of Herpes Zoster Vaccines [Internet]. Public Health Agency of Canada; 2018 Jun. Available from: https://www.canada.ca/content/dam/phac-aspc/documents/services/publications/healthy-living/updated-recommendations-use-herpes-zoster-vaccines-eng.pdf

40. An Advisory Committee Statement (ACS) National Advisory Committee on Immunization (NACI) - Guidance on COVID-19 vaccine booster doses: Initial considerations for 2023 [Internet]. Public Health Agency of Canada; 2023 Jan. Available from: https://www.canada.ca/content/dam/phac-aspc/documents/services/immunization/national-advisory-committee-on-immunization-naci/guidance-covid-19-vaccine-booster-doses-initial-considerations-2023/guidance-covid-19-vaccine-booster-doses-initial-considerations-2023.pdf

41. Rasul G. A Framework for Improving Policy Priorities in Managing COVID-19 Challenges in Developing Countries. Front Public Health [Internet]. 2020 Oct 14;8:589681. Available from: http://dx.doi.org/10.3389/fpubh.2020.589681

42. Stratil JM, Voss M, Arnold L. WICID framework version 1.0: criteria and considerations to guide evidence-informed decision-making on non-pharmacological interventions targeting COVID-19. BMJ Glob Health [Internet]. 2020 Nov;5(11). Available from: http://dx.doi.org/10.1136/bmjgh-2020-003699

Classified as ECDC NORMAL

43. Van 't Veer P, Grammatikaki E, Matthys C, Raats MM, Contor L. EURRECA-Framework for Aligning Micronutrient Recommendations. Crit Rev Food Sci Nutr [Internet]. 2013;53(10):988–98. Available from: http://dx.doi.org/10.1080/10408398.2012.742857

44. Dhonukshe-Rutten RAM, Timotijevic L, Cavelaars AEJM, Raats MM, de Wit LS, Doets EL, et al. European micronutrient recommendations aligned: a general framework developed by EURRECA. Eur J Clin Nutr [Internet]. 2010 Jun;64 Suppl 2:S2–10. Available from: http://dx.doi.org/10.1038/ejcn.2010.55

45. Krahn M, Miller F, Bayoumi A, Brooker AS, Wagner F, Winsor S, et al. DEVELOPMENT OF THE ONTARIO DECISION FRAMEWORK: A VALUES BASED FRAMEWORK FOR HEALTH TECHNOLOGY ASSESSMENT. Int J Technol Assess Health Care [Internet]. 2018 Jun;34(3):290–9. Available from: http://dx.doi.org/10.1017/S0266462318000235

46. Almeida ND, Mines L, Nicolau I, Sinclair A, Forero DF, Brophy JM, et al. A Framework for Aiding the Translation of Scientific Evidence into Policy: The Experience of a Hospital-Based Technology Assessment Unit. Int J Technol Assess Health Care [Internet]. 2019 Jan;35(3):204–11. Available from: http://dx.doi.org/10.1017/S0266462319000254

47. Veglia A, Pahwa M, Demers PA. Establishing a Policy Framework for the Primary Prevention of Occupational Cancer: A Proposal Based on a Prospective Health Policy Analysis. Saf Health Work [Internet]. 2017 Mar;8(1):29–35. Available from: http://dx.doi.org/10.1016/j.shaw.2016.07.001

48. Votruba N, Grant J, Thornicroft G. The EVITA framework for evidence-based mental health policy agenda setting in low- and middle-income countries. Health Policy Plan [Internet]. 2020 May 1;35(4):424–39. Available from: http://dx.doi.org/10.1093/heapol/czz179

49. Votruba N, Grant J, Thornicroft G. EVITA 2.0, an updated framework for understanding evidence-based mental health policy agenda-setting: tested and informed by key informant interviews in a multilevel comparative case study. Health Res Policy Syst [Internet]. 2021 Mar 10;19(1):35. Available from: http://dx.doi.org/10.1186/s12961-020-00651-4

50. Shafaghat T, Bastani P, Nasab MHI, Bahrami MA, Montazer MRA, Zarchi MKR, et al. A framework of evidence-based decision-making in health system management: a best-fit framework synthesis. Arch Public Health [Internet]. 2022 Mar 29;80(1):96. Available from: http://dx.doi.org/10.1186/s13690-022-00843-0

51. Canfell OJ, Davidson K, Sullivan C, Eakin EE, Burton-Jones A. PREVIDE: A Qualitative Study to Develop a Decision-Making Framework (PREVention decIDE) for Noncommunicable Disease Prevention in Healthcare Organisations. Int J Environ Res Public Health [Internet]. 2022 Nov 18;19(22). Available from: http://dx.doi.org/10.3390/ijerph192215285

52. Guide to Community Preventive Services. Methods Manual for Community Guide Systematic Reviews [Internet]. [cited 2023 Jun 2]. Available from: https://www.thecommunityguide.org/pages/methods-manual.html

53. HIV Prevention and Control: Partner Services to Increase HIV Testing [Internet]. Community Preventive Services Task Force; 2021 Jul. Available from: https://www.thecommunityguide.org/findings/hiv-prevention-partner-services-interventions-increase-hiv-testing.html

54. CPSTF Findings for Increasing Vaccination [Internet]. Community Preventive Services Task Force; 2016 Feb. Available from: https://www.thecommunityguide.org/pages/task-force-findings-increasing-vaccination.html

55. Rehfuess EA, Akl EA. Current experience with applying the GRADE approach to public

health interventions: an empirical study. BMC Public Health [Internet]. 2013 Jan 8;13:9. Available from: http://dx.doi.org/10.1186/1471-2458-13-9

56. Zähringer J, Schwingshackl L, Movsisyan A, Stratil JM, Capacci S, Steinacker JM, et al. Use of the GRADE approach in health policymaking and evaluation: a scoping review of nutrition and physical activity policies. Implement Sci [Internet]. 2020 May 24;15(1):37. Available from: http://dx.doi.org/10.1186/s13012-020-00984-2
